# The Potential of Indonesian Heterobranchs Found around Bunaken Island for the Production of Bioactive Compounds

**DOI:** 10.3390/md15120384

**Published:** 2017-12-07

**Authors:** Katja M. Fisch, Cora Hertzer, Nils Böhringer, Zerlina G. Wuisan, Dorothee Schillo, Robert Bara, Fontje Kaligis, Heike Wägele, Gabriele M. König, Till F. Schäberle

**Affiliations:** 1 Institute for Insect Biotechnology, Justus-Liebig-University Giessen, 35392 Giessen, Germany; Katja.M.Fisch@agrar.uni-giessen.de (K.M.F.); nils.boehringer@uni-bonn.de (N.B.); Zerlina.G.Wuisan@bio.uni-giessen.de (Z.G.W.); 2 Institute for Pharmaceutical Biology, Rheinische Friedrich-Wilhelms-University Bonn, 53115 Bonn, Germany; s6coherz@uni-bonn.de; 3 Centre of Molecular Biodiversity, Zoological Research Museum Alexander Koenig, 53113 Bonn, Germany; doro.schillo@gmail.com (D.S.); H.Waegele@leibniz-zfmk.de (H.W.); 4 Faculty of Fisheries and Marine Science, Sam Ratulangi University, Manado 95115, Indonesia; robert.bara@unsrat.ac.id (R.B.); fontjekaligis@yahoo.com (F.K.); 5 German Center for Infection Research, Partner Site Bonn-Cologne, 53115 Bonn, Germany

**Keywords:** bioactivity, biodiversity, natural products, sea slug

## Abstract

The species diversity of marine heterobranch sea slugs found on field trips around Bunaken Island (North Sulawesi, Indonesia) and adjacent islands of the Bunaken National Marine Park forms the basis of this review. In a survey performed in 2015, 80 species from 23 families were collected, including 17 new species. Only three of these have been investigated previously in studies from Indonesia. Combining species diversity with a former study from 2003 reveals in total 140 species from this locality. The diversity of bioactive compounds known and yet to be discovered from these organisms is summarized and related to the producer if known or suspected (might it be down the food chain, de novo synthesised from the slug or an associated bacterium). Additionally, the collection of microorganisms for the discovery of natural products of pharmacological interest from this hotspot of biodiversity that is presented here contains more than 50 species that have never been investigated before in regard to bioactive secondary metabolites. This highlights the great potential of the sea slugs and the associated microorganisms for the discovery of natural products of pharmacological interest from this hotspot of biodiversity.

## 1. Introduction

Eat or be eaten—many mechanisms have evolved during millions of years to prevent organisms falling into the second group. Most mollusks (soft-bodied marine organisms) use the mechanical properties of a shell to protect themselves from predators. However, loss of the shell in several mollusk groups indicates that a shell is an obstruction for some lifestyles. Hence, as a result new strategies have to be developed to protect these animals against predators. Octopuses and squids are responsive and fast, thereby able to escape potential predators by speed. Gastropoda (snails and slugs) instead must rely on other mechanisms to survive common predators like fish, crabs or echinoderms. Beside camouflage, they mostly use chemicals for protection, either by incorporation and use of cnidocysts and the toxins therein from their prey, or by sequestration of other chemical weapons. It has been shown that sea slugs, e.g., *Phyllodesmium* species, apply the chemical diversity of their specific food source as defensive mechanisms [1]. Compounds which have been isolated from the coral food were obtained in much higher amounts from the slugs, indicating the accumulation of these metabolites. Further, de novo synthesis of defensive metabolites by slugs is reported [2,3,4]. However, it has to be kept in mind that compounds may also be produced by bacteria associated with the sea slug or their food, e.g., algae, sponges and bryozoa. This was exemplified by dolastatin 10 (**1**), first described from the anaspidean *Dolabella auricularia* and subsequently from cyanobacteria [5,6,7] on the algal food.

The entity of natural products isolated from marine mollusks is intriguing and would justify comprehensive books and review articles and many natural products have been reviewed previously in such [8,9]. This review however focusses on sea slug-derived molecules for which biological activity is proven to show the potential for human use or for understanding ecological context. Furthermore, a regional focus is applied, i.e., the species diversity of sea slugs found at field trips in Bunaken National Park (BNP; North Sulawesi, Indonesia, Table 1 in Section 3.1) forms the base to show the status of investigation and the diversity of bioactive compounds to be expected from this biodiversity hotspot. However, the natural products described from the same species in previous literature might be isolated from specimens collected at other locations around the globe. For comparison, this origin is always given. If a producer of a bioactive metabolite, e.g., the respective food source or associated bacteria, is known or suspected, this is pointed out. A summary of the species with previously indentified bioactive compounds and their putative origin is provided at the end of the review (Table 2).

## 2. Scope of the Review and Methodology Applied

The review focusses on bioactive compounds from heterobranchs and additionally applies a regional focus. The expedition to BNP, which forms the basis for the species list used for this review (Table 1), took place in August 2015. Collecting areas comprised 16 sites with the focus on Bunaken Island (11 sites), three sites around Manado Tua, one site at Siladen Island and one site opposite to Bunaken Island along the mainland of North Sulawesi (Tiwoho), not belonging to the BNP. Although some sites were revisited (e.g., during the night), the collecting area rarely overlaped with previous visits. Overall, 18 dives (16 during daytime and two dives during night) with 3–5 divers were performed, and several hours were spent snorkelling at the dive spots. One dive usually lasted 60 min, with a few exceptions of up to 120 min, rendering the total amount of underwater searching time to about 100 to 120 h manpower. Prior experience in searching and collecting sea slugs under water varies between the divers from extremely high (one diver with daily experience for several years), up to medium (two divers) and marginal experience (two divers).

Sea slugs were always collected directly from substrate in the field by scuba diving or by snorkelling. We documented the collected species by under-water photography as well as close up pictures and identification was checked by consulting most recently published identification books [10,11,12], by seaslugforum (www.seaslugforum.net), or primary literature (e.g., [13,14]). Validity of names was checked with the help of the World Register of Marine Species (www.marinespecies.org) and with Gosliner et al. (2015) [12]. Systematics in marine heterobranchs has changed considerably in the last decade and therefore old and new names are given for better understanding, especially when older literature is involved. It has to be mentioned that no substrate samples (algae, sediment or coral rubble) were collected. Thus, tiny and interstitial heterobranchs are certainly missing. However, these especially small species do not represent good starting material for chemical investigations, since the material would be extremely limiting. The metadata of the animals will be available in Diversity Collection (Diversity Workbench). Usually, a small piece of the animals was taken and stored in 96% EtOH for future barcoding, which is currently work in progress. All material was collected with necessary permissions according to the Nagoya Protocol.

This review focusses on bioactive compounds, i.e., compounds for which a bioactivity has been previously tested and confirmed. As it might be the case that bioactivity was detected later than the initial decription of the natural product, after a search for each detected heterobranch genus name using Pubmed (https://www.ncbi.nlm.nih.gov/pubmed), the names of the identified compounds were also used as a search term in the same database. Papers of interest were followed up in both directions, i.e., in literature they cite and in literature these papers were cited in using Web of Science, especially to make sure structural amendments and more specific literature has not been overlooked. Structurally related non-active compounds, reported from the same species were included in some cases to show structural diversity, e.g., sesquiterpenes from *Phyllidia* species. Of course, it might be the case that some compounds possess any bioactivity which is not identified until now. However, for the purpose of this review, we focus on the compounds with established bioactivity, including anticancer, antimicrobial, anthelminthic, antifouling, anti-inflammatory, ichthyotoxic and fish deterrent activity.

## 3. Natural Products with Biological Activity from Heterobranchia Families Found near Bunaken

### 3.1. Biodiversity of Sea Slugs Found in the Bunaken National Park Survey

Studies on marine heterobranchs from specific Indonesian areas are rare. Usually records are listed in general studies or identification books. Only a few studies assessing biodiversity of these slugs are focussing on one locality. The most extensive studied area of Indonesia is Ambon (part of the Maluku Islands) [13,15,16]. The second most studied is probably Bali; also included in a recent sea slug census in Lembeh (North Sulawesi) and Bali finding only 8 species at both locations and 51 unique to Lembeh vs. 27 to Bali [17]. However, the slug species in the census were identified only by field pictures and not documented in the scientific literature [17]. BNP, as the targeted area in the most Northern region of Indonesia, has only been surveyed once previously in order to assess molluscan diversity [18]. In this former study about 80 marine heterobranch species were recorded. During our collection in 2015, again around 80 heterobranch species were sampled (Table 1). However, only 21 species were recorded in both surveys (Kaligis et al. in revision). Thus, the combined species number recorded from BNP is nearly 140 species, including several undescribed species. The group known for their bioactive compounds, the Anthobranchia, are especially well represented now with more than 30 species (including probably three undescribed species) (Kaligis et al. in revision, Table 1). This indicates BNP as a study area of high species richness with a great potential to find species, not only in the pharmaceutically well studied group of Anthobranchia, but also in other less known groups, like the Cephalaspidea. According to the species composition that is certainly related to the coral habitats around BNP, some groups that are also known for their pharmaceutical importance are less represented. This mainly refers to the groups Sacoglossa and Anaspidea, which need habitats dominated by rich algal communities [19]. However, few representatives of these groups (even new species) were collected (Table 1). In the following review, all families that were represented by members in our collection are discussed in the light of bioactive compounds, be it already described compounds from that specific species in other localities), or by indicating their potential based on bioactive compounds in related species (see Table 1 and Table 2). Only three studies report bioactive compounds from Indonesian heterobranchs, i.e., *Pleurobranchus forskalii*, *Chromodoris lochi* and *Phyllidia varicosa*. The unique peptide found in *P. forskalii* from Manado (Indonesia) (see Section 3.5.1 and Table 2) shows that even relatively well investigated species are of high interest, due to the intraspecific variances in compound composition in regard to geographic distribution and localities. Of special interest are also Heterobranchia families which have no reported bioactive compounds such as Diaphanidae, Goniodorididae, Gymnodoridae and Eubranchidae, which most likely have not been investigated so far.

### 3.2. Anaspidae (Sea Hares)

#### Aplysiidae

**Figure 1:**
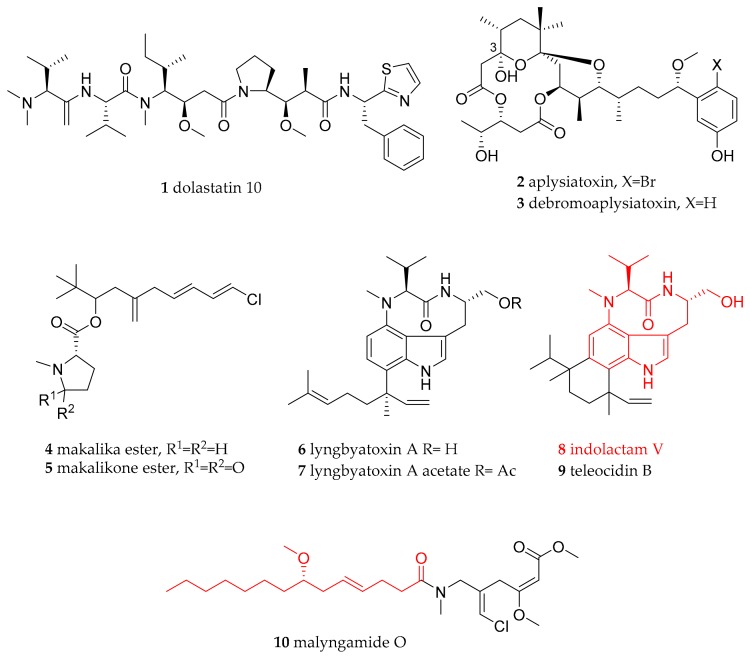
Biologically active natural products isolated from *Stylocheilus* species (including indolactam V and teleocin B for structure comparison).

*Stylocheilus striatus* from the Aplysiidae family was found near Bunaken. Since we did not find specimens of the *Aplysia* genus and there is a comprehensive review on secondary metabolites of these sea slugs [20] they have not been further discussed here. Additionally, for the same reason *Dolabella* metabolites like the famous dolastatin 10 (1) are not discussed [21]. The focus here is on the genus *Stylocheilus*. Even though the toxicity of sea hares has been known for a long time, it took until 1974 until the so-called ether-soluble toxin of *S. longicauda* was identified as an oily mixture consisting of aplysiatoxin (2) and debromoaplysiatoxin (3) (Figure 1) [22]. The toxicity of this mixture was determined in mice, i.e., LD_100_ 0.3 mg/kg, ip mouse [23,24]. The toxins 2 and 3 were labile above pH 7 and below pH 4, due to a phenolic hydroxyl and the tertiary hydroxyl at C-3. Altogether, including the artifacts ensuing from the experimental conditions, eight derivatives were isolated. The toxins 2 and 3 have later been isolated from the cyanobacterium *L. majuscula* which is a food source of *S. longicauda* [25] and a biosynthesis via a polyketide biosynthesis pathway is most likely. Both are potent protein kinase C (PKC) activators and research is ongoing to develop analogues as anticancer lead structures, based on their anti-proliferative activity, but removing any tumour-promoting activities [26].

A later study on *S. longicauda* from Hawaii revealed the chlorinated metabolites makalika ester (4) and makalikone ester (5) (Figure 1) [27]. Using high-resolution mass spectrometry the molecular formula of 4 was determined to be C_19_H_30_ClNO_2_, while 5 was carrying one additional *O*-atom, resulting in the molecular formula C_19_H_28_ClNO_3_. The structures were elucidated by NMR analysis and finally the stereochemistry of the *N*-proline residue was determined by hydrolysis of the ester. Following purification of the amino acid, its optical rotation was measured and confirmed the stereochemistry by comparison with L-*N*-methylproline. Compound 5 showed moderate activity against the cancer cell lines P388, A549, and HTB38, i.e., IC_50_ in the range of 2.5–5 µg/mL [27]. The *tert*-butyl functionality present in 4 and 5 is uncommon in natural product chemistry; however, it has been identified before in isolates from the cyanobacteria *L. majuscula* and *L. bouillonii* [28,29,30,31]. A recurring feature in these molecules is the connection of the *tert*-butyl to the ester linkage and the *N*-methyl via an oxymethine carbon [27]. This structural connection of the molecules is reflected in the connection of *S. longicauda* with *L. majuscula*, i.e., the sea slug is known to feed on the latter regardless of its toxic and unpalatable compounds produced. More compounds of cyanobacterial origin were found in *Stylocheilus* species in particular lyngbyatoxin A (**6**) and lyngbyatoxin A acetate (**7**) (Figure 1), as well as the malyngamides (Figure 1). The structure elucidation of **6** was first reported in detail from *L. majuscula* and its toxicity determined to mice (LD_100_ = 0.3 mg/kg) and *Poecilia vittata* (baitfish), killing all fish within 30 min at 0.15 µg/mL [32]. Compound **6** also showed inflammatory activity and acts as a tumour-promoter [32]. The authors were able to deduce the structure of 6 using NMR by comparison to the reported values of the (−)-indolactam V (**8**) part of teleocidin B (**9**) a toxin produced by various *Streptomyces* species [32]. The biosynthetic genes for **6** and **9** have been identified revealing a mixed non-ribosomal peptide tepenoid biosynthesis [33,34]. The acetylated derivative **7** isolated from *S. longicauda* from Hawaii showed very potent toxicity with IC_50_ values against the cancer cell lines tested, i.e., IC_50_ 0.05 µg/mL [27].

In 2000, analysis of *S. longicauda* from Hawaii enlarged the arsenal of known malyngamides reported before from cyanobacterial species [32,35,36]. The malyngamide O (**10**) (Figure 1) was isolated from the freeze-dried sea slug, known to feed on *L. majuscula*. In **10**, the typical C_14_ acid (in Figure 1 shown red) of malyngamides is linked to an acyclic amine. Toxicity tests of **10** with the cancer cell lines P388, A549, and HT29 gave values of IC_50_ 2 µg/mL.

Other sea slugs also use *L. majuscula* as a food source, e.g., *S. striatus*, *Bursatella*, and the cephalaspidean *Diniatys dentifer* are described as grazers upon this toxic cyanobacterium. In certain tissues the levels of **3** and **6** reached high values indicating the bioaccumulation of these compounds in special tissues [37].

### 3.3. Sacoglossa

#### 3.3.1. Caliphyllidae

**Figure 2:**
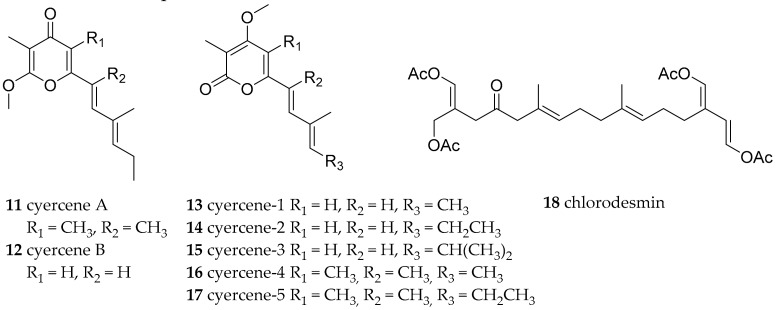
Bioactive compounds from *Cyerce* species.

*Cyerce* sp. 4 and sp. 2 (perhaps *Cyerce bourbonica*) were found at the BNP. No natural products have been reported from this species yet, but the α- and γ-pyrones cyercene A (**11**) and B (**12**) and 1–5 (**13**–**17**) (Figure 2) were isolated from *Cyerce cristallina* from the Mediterranean Sea and exhibited high ichthyotoxicity against *Gambusia affinis*, with cyercene A (**11**), cyercene-3 (**15**) and -4 (**16**) being very toxic (10 μg/mL). These metabolites are de novo synthesized by *C. cristallina* via the polypropionate pathway [2,38]. A possible role in ceratal growth was hypothesized, as well as a protective function against sunlight-induced damage. Further, the absence of α- and γ-pyrones from the family member *Caliphylla mediterranea* was reported. Chlorodesmin (**18**, Figure 2), a modified diterpene known from the green algae *Chlorodesmis fastigiata* was isolated from *Cyerce nigricans* obtained near Lizard Island (Australia) ([39] and references herein). Compound **18** has shown antibacterial and antifungal activity, but failed to show fish deterrent properties against *Thalassoma lunare* or a small wrasse species [39,40].

#### 3.3.2. Oxynoidae

**Figure 3:**
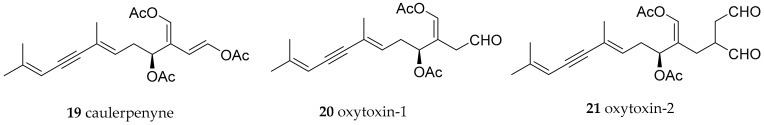
Caulerpenyne and related metabolites from Oxynoidae mollusk.

*Lobiger* sp. 1 and *Lobiger viridis* have been found at BNP. A detailed investigation of the defensive relationship between the green alga *Caulerpa prolifera* and three Sacoglossan predators showed that *Lobiger serradifalci* transformed the algal metabolite caulerpenyne (**19**) mainly to oxytoxin-1 (**20**), whereas another family member *Oxynoe olivacea* transformed it further and also contained also oxytoxin-2 (**21**) (Figure 3) [41]. Both compounds were deterrent against marine fishes and toxic against *Gambusia affinis*. Caulerpenyne is a known biotoxin from *Caulerpa taxifolia*, which affects several cellular and molecular targets and can cause neurological disorders [42,43].

#### 3.3.3. Plakobranchidae

*Elysia asbecki* and at least 3 unidentified *Elysia* species were collected at BNP. Further, *Thuridilla albopustulosa*, *Thuridilla flavomaculata*, *Thuridilla gracilis* and *Thuridilla lineolata* were found.

To date, manifold chemical studies on *Elysioidean* species collected from distinct geographic areas from the Indo-Pacific Ocean to the Mediterranean Sea have been published [44,45,46,47,48,49]. A chemical marker for a selected group of *Elysia* sacoglossans including *E. chlorotica* and *E. viridis* are γ-pyrone polypropionates, which have been reported from different geographical areas in the world [47,50]. These polypropionates, e.g., elysione (**22**) are de novo biosynthesized by the slugs and might complement the photoprotective role of algal chloroplast pigments in a photolytic habitat, since the biosynthesis of these molecules is influenced by light irradiation, justifying preservation of this pathway in Elysoidean molluscs [47,50,51].

**Figure 4:**
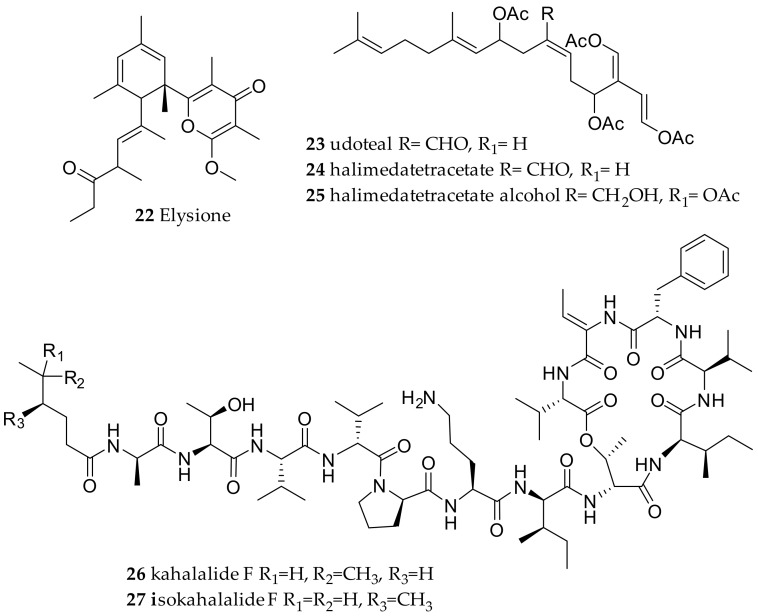
Bioactive compounds from *Elysia* species.

Algal derived sesquiterpenoids such as **19**, **20** and **21** have also been found in several *Elysia* species, as well as the diterpenoid udoteal (**23**) with associated antibacterial and antifungal activity having been isolated from *E. translucens* [3,40]. The ability to chemically modify ingested algal terpenoids has also been reported from *E. halimedae* transforming the halimedatetracetate (**24**) to halimedatetracetate alcohol (**25**) (Figure 4) [52].

Most important, from a pharmacological point of view, was the isolation of dietary algal metabolites including a large family of structurally unrelated depsipeptides, called kahalalides, from *E. rufescens*, *E. ornata*, *E. grandifolia* and their algal diet *Bryopsis pennata* [46,48]. These cyclodepsipeptides exhibit highly diverse biological activities, such as antiviral, antifungal, antileishmanial, cytotoxic and immunosuppressive properties [46,48,53,54]. The most important representative is kahalalide F (**26**), which is the largest and most biologically active compound of these cyclic peptides [46,48]. Compound **26** occurs naturally as a mixture with its isomer isokahalalide F (**27**), which also shows interesting biological activities [55,56]. It was proposed that **26** and **27** might be of bacterial origin, with *Mycoplasma* spp. and *Vibrio* spp. as possible producers being the most abundant bacterial groups affiliated with *E. rufescens* and its mucus [57].

**Figure 5:**
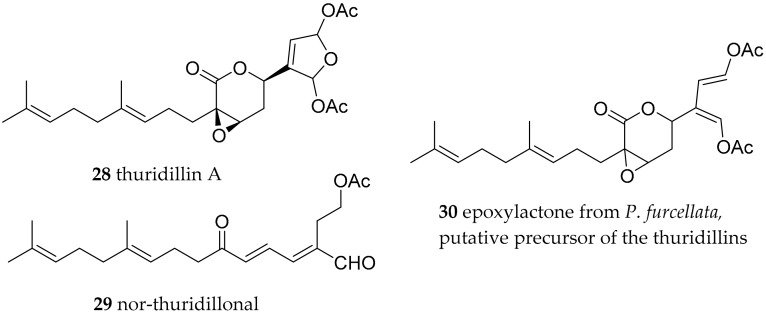
Thuridillins and the related epoxylactone from *Pseudochlorodesmis furcellata*, a green algae (Chlorophyta).

First reports from a *Thuridilla* species were performed in Italy on *Thuridilla hopei* [58]. Thereby, the diterpenoid thuridillins were isolated, possessing a central α,β-epoxy-δ-lactone ring which is substituted by an uncyclized or cyclized isoprenoid chain and a 2,5-diacetoxy-2,5-dihydrofuran unit. *T. splendens* from Australia yielded new thuridillins, together with thuridillin A (**28**, Figure 5) [59]. The separation of the various thuridillins was challenging, but thuridillin-related aldehydes isolated from a Mediterraneam *T. hopei* were even more unstable. The samples decomposed during NMR measurements using CDCl_3_ as solvent. Subsequent analysis in C_6_D_6_ was advantageous. Nor-thuridillonal (**29**) proved to be the epoxylactone (**30**, Figure 5) from the algae *Pseudochlorodesmis furcellata* [60]. It shows the same carbon scaffold and possesses significant feeding deterrent properties, thus was implicated as the precursor diterpene of the thuridillins in *T. hopei*.

### 3.4. Cephalaspidea

#### 3.4.1. Aglajidae

At the BNP we found an as yet unidentified specimen belonging into the Aglajidae family as well as *Chelidonura amoena*, *Chelidonura hirundinina* and *Odontoglaja guamensis*. No bioactive compounds have reported for any of these species. However, bioactive compounds have been isolated from *Philinopsis* and *Navanax* species within the same family.

**Figure 6:**
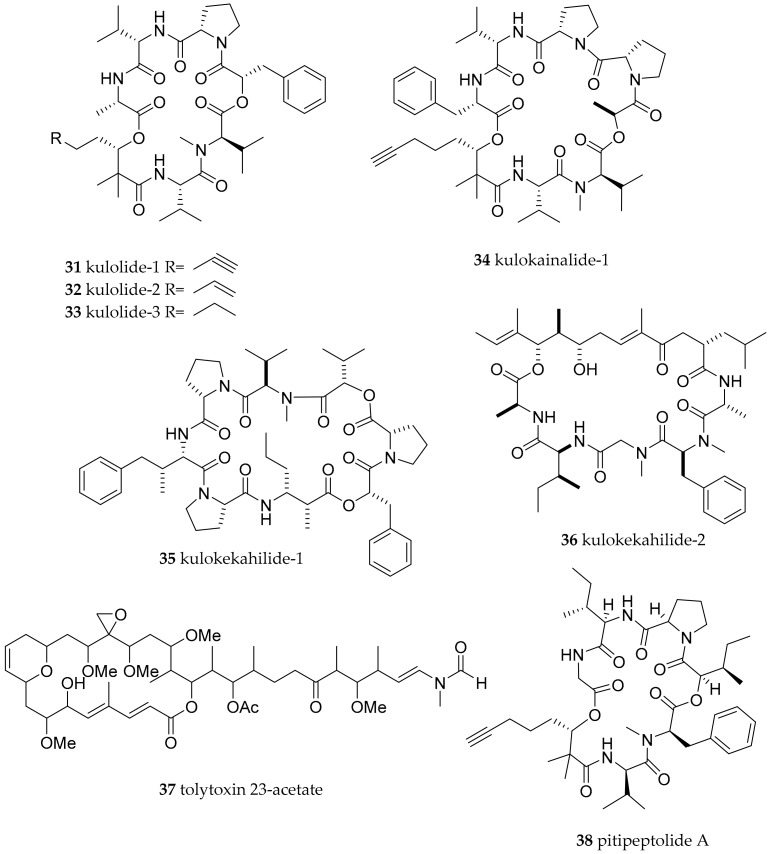
Biologically active natural products from molluscs of the Aglajidae family and the cyanobacterial pitipeptolide A.

Kulolide-1 (**31**, Figure 6), a cyclic depsipeptide with potent cytotoxicity and anti-tumour activity (0.7 µg/mL against L-1210 and 2.1 µg/mL against P388 cell line) was isolated from an Hawaiean *Philinopsis speciosa* specimen [61]. Further, analysis of Hawaiian specimen revealed the presence of more peptides of the kulolide family such as kulolide-2 (**32**), kulolide-3 (**33**), kulokainalide-1 (**34**) and the unusual didepsipeptides kulokekahilide-1 (**35**) and kulokekahilide-2 (**36**, Figure 6) [62,63,64]. Moderate cytotoxicity was reported for **34** and **35,** but potent cytotoxicity as well as selective cytotoxicity for **36** (P388, SK-OV-3, MDA-MB-435, and A-10 with IC_50_ values of 4.2, 7.5, 14.6, and 59.1 nM, respectively, and the A-10 cell line not transformed). Additionally, the macrolide tolytoxin 23-acetate (**37**) was suspected to be responsible for some of the activity measured for **34** and related peptides [63]. Striking structural similarity between molecules from *P. speciosa* and from cyanobacteria implies that their biosynthetic origin lies in cyanobacteria and the molecules from the slugs are of dietary origin. The transfer is most likely mediated via herbivorous molluscs like the sea hares *Stylocheilus longicauda* and *Dolabella auricularia* which in turn were readily eaten by *P. speciosa* in a feeding experiment [63]. Furthermore, the same study confirmed the presence of **31** in *S. longicauda*. The capability of sea slugs to handle toxins produced by cyanobacteria is also shown by the fact that pitipeptolide A (**38**, Figure 6), a kulolide family depsipeptide produced by the cyanobacterium *Lyngbya majuscula*, was deterrent to various small invertebrates which usually can tolerate algal chemical defensive molecules; but it did not deter feeding of *Stylocheilus striatus* [65], which in turn can be prey for sea slugs of the Aglajidae family.

The underlying biosynthetic pathway for the kulolide is most likely a non-ribosomal peptide synthetase, also incorporating unusual non-proteinogenic amino acids. The kulolide type molecules can be further subdivided into subgroups with distinct changes in biological activity [66].

**Figure 7:**
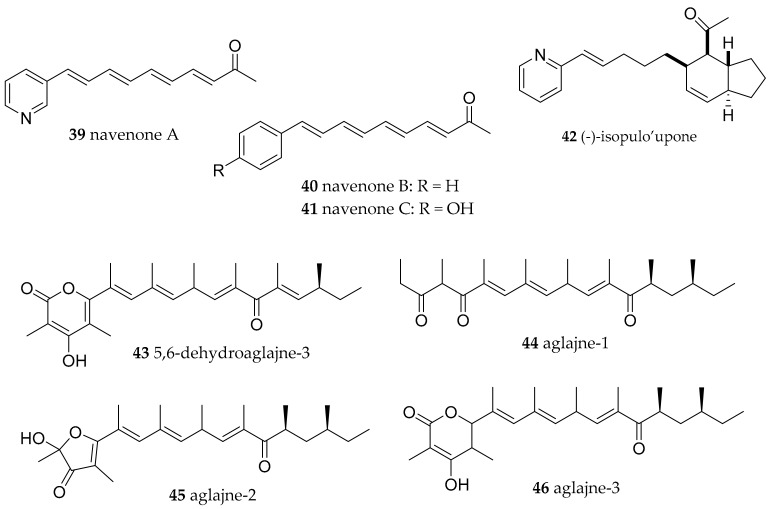
Further biologically active natural products from molluscs of the Aglajidae family.

Another member of the Aglajidae family, *Navanax inermis* is known to be a rich source of bioactive compounds. The first natural products found in Pacific *N. inermis* specimens were the navenones A-C (**39**–**41**) serving as pheromones [67]. Furthermore, the very ichthyotoxic isopulo’upone (**42**) and polypropionate 5,6-dehydroaglajne-3 (**43**) were found in Pacific specimen [68]. The toxic (*Artemia salina* LD_50_ < 35 ppm) polypropionates aglajne-1 (**44**), aglajne-2 (**45**) and aglajne-3 (**46**) (Figure 7) were isolated from the Mediterranean family member *P. depicta* [69]. Compound **46** was also very ichthyotoxic against *Gambusia affinis*. *P. depicta* and *N. inermis* are carnivorous and feed on *Bulla* species. Again, a detailed investigation in compounds contained by prey and predator revealed a good relationship and suggest they are of dietary origin [69,70].

#### 3.4.2. Gastropteridae

*Sagaminopteron psychedelicum*, *Siphopteron tigrinum*, *Siphopteron* cf *ladrones*, *Siphopteron brunneomarginatum*, *Siphopteron nigromarginatum* and *Siphopteron* spec have been found at BNP. Until now, no natural products have been reported from *Siphopteron* species.

**Figure 8:**
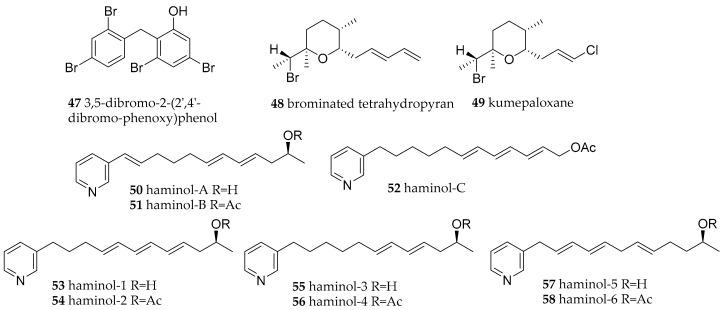
Bioactive metabolites from sea slugs of the Gastropteridae and Haminoidae families.

*Sagaminopteron nigropunctatum* and *S. psychedelicum* seemed to have chosen different strategies to repel or hide from predators. *S. psychedelicum*’s phenotypic appearance is eye-catching. Such a warning coloration is typical for many opisthobranchs, which use chemical molecules to defend themselves. *S. nigropunctatum* instead is highly cryptic and hard to spot on the sponge these two species can be found on. Analysis of the natural products derived from these sea slugs revealed that both species possess polybrominated diphenyl ethers (BDEs), especially 3,5 dibromo-2-(2′,4′-dibromo-phenoxy)phenol (**47**, Figure 8) which can also be found within their host sponge *Dysidea granulosa* [71]. Compound **47** was detected in the mantle of the slugs in the same concentration as in the sponge, e.g., 2–4%. However, in the parapodia of the slugs the compounds showed a concentration of approximately 8–10%, indicating that the sea slugs concentrate the BDEs in these appendages. Antifouling activity of **47** has been evaluated against marine bacteria, a diatom, barnacle larvae and mussel juveniles and found to be highly active, but non-toxic [72].

#### 3.4.3. Haminoeidae

Two different species of *Haminoea* have been identified near Bunaken. In *H. cyanomarginata* from Greece, the brominated tetrahydropyran (**48**) was isolated [73], and was reported also in the congeneric species *H. cymbalum* from Indian coasts and stated to be previously isolated from an Australian sponge. It is structurally related to kumepaloxane (**49**) found in *H. cymbalum* from Guam [74]. Compound **48** proved to be highly toxic to the mosquito fish *G. affinis* at the concentration of 1 ppm [73]. It also produced a strong food rejection in the generalist marine shrimp *Palaemon elegans*.

*Haminoea* species are known to contain 3-alkylpyridines named haminol A–C (**50**–**52**) and haminol 1–6 (**53**–**58**) (Figure 8) [75], which are similar to the navenones in *Navanax* species (see Figure 7). Haminols act as alarm pheromones and have been tested for antifouling properties; especially **54** showing good anti-settlement activity against larvae of the barnacle *Amphibalanus amphitrite* and low toxicity [76]. Feeding studies with labelled nicotinic acid methyl ester and acetate showed de novo synthesis of **54** in the Mediterranean mollusc *H. orbignyana*, suggesting nicotinic acid as an unusual PKS starter unit and the side chain polyketide derived [4]. However, these findings do not rule out that associated microorganisms are the actual producers.

Two polypropionates similar to 5,6-dehydroaglajne-3 (**43**), have also been isolated from the Mediterranean *H. fusari* after methylation together with the bioactive haminols (**53**–**58**), but no bioactivity has been examined [77].

### 3.5. Pleurobranchomorpha

#### 3.5.1. Pleurobranchidae

**Figure 9:**
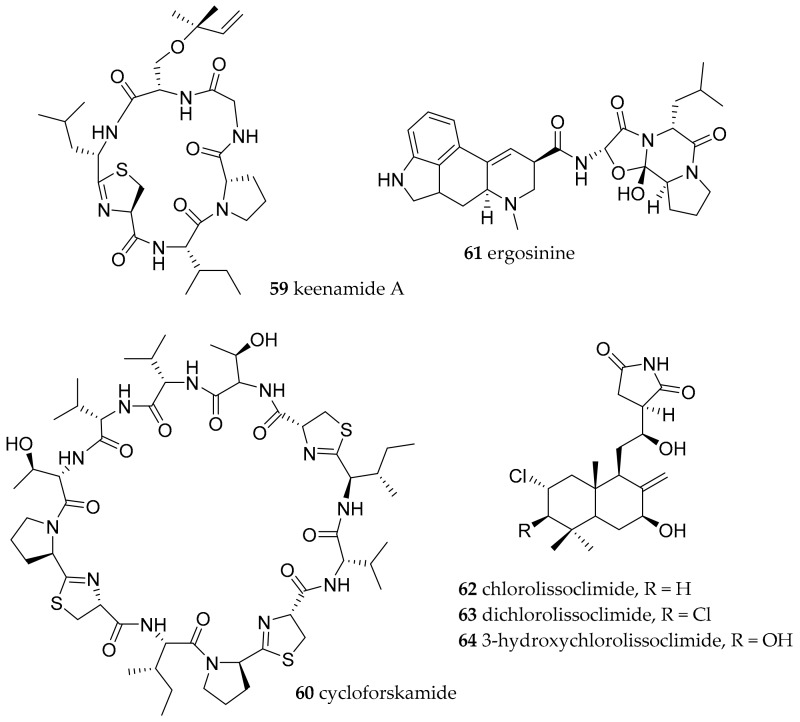
Biologically active natural products isolated from *Pleurobranchus* species.

*Pleurobranchus forskalii* was collected from BNP. The first cyclic peptide isolated from *Pleurobranchus forskalii* collected from Manado (the town vis-à-vis from Bunaken Island) was keenamide A (**59**) (Figure 9) [78]. This cytotoxic cyclic hexapeptide comprising of a thiazoline and an isoprene residue was tested against several tumor cell lines and showed IC_50_ values in the range from 2.5 µM (against P388, A549, and MEL20) to 5 µM (against HT29). A further macrocyclic peptide isolated from *P. forskalii* is cycloforskamide (**60**), a dodecapeptide (Figure 9) [79]. It was isolated by chromatography techniques and its molecular formula is C_54_H_86_N_12_O_11_S_3_ showing three thiazoline heterocycles. Using chiral-phase gas chromatography, the authors succeeded to deduce the absolute configuration. The presence of three D-amino acids point towards a non-ribosomal peptide synthetase (NRPS) system as the biosynthetic basis for this cyclic peptide. It might be diet or symbiont-derived. However, its similarity to cyanobactins also points towards cyanobacterial origin. Cyanobactins are an example of symbiont derived (ribosomally synthesised) cyclic peptides containing thiazoline moieties found in several sponges, but biosynthetic genes are encoded in symbiotic cyanobacteria [80]. The peptide **60** showed cytotoxicity against the murine leukemia cell-line P388 (IC_50_ of 5.8 µM)

Ergosinine (**61**) was isolated from *P. forskalii* collected from Ishigaki Island, Japan [81]. This was the first time such an alkaloid had been isolated from marine environments, which was until then only described from terrestrial higher plants and fungi. The authors speculate that this indicates the accumulation of ergopeptines in macroorganisms living in marine habitats around the coast. Toxic effects of ergot alkaloids are known since medieval times and caspase-3-activation have also been determined [82].

From the *Pleurobranchus* species *P. albiguttatus* and *P. forskalii*, collected in the Philippines, chlorinated diterpenes were isolated [83]. Similar diterpenes had been reported before from *Lissoclinum* species of the taxon Ascidiacea. This finding gave a direct hint towards the original source of the diterpenes to be the prey of *Pleurobranchus*. Chlorolissoclimide (**62**) and dichlorolissoclimide (**63**) were reported to be potent cytotoxins and 3β-hydroxychlorolissoclimide (**64**, Figure 9) exhibited solid tumor selectivity.

Bioassay-guided fractionation for protein synthesis inhibitors also yielded **62** and **63** as the active components [84]. The IC_50_ values determined for **62** and **63** were 0.7 µM and 1.25 µM, respectively. An antibacterial effect was not detected. However, it was revealed that lissoclimides block translation elongation by inhibiting translocation, which results in an accumulation of ribosomes on mRNA.

### 3.6. Nudibranchia

The richest diversity among the Heterobranchs at BNP were found from the shell-less Nudibranchia families listed below.

#### 3.6.1. Aegiridae

**Figure 10:**
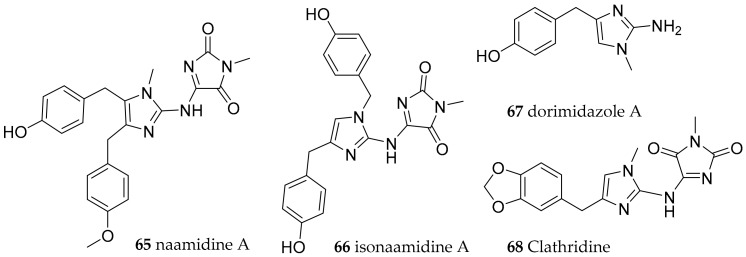
Biologically active natural products isolated from molluscs of the Aegiridae family.

During the field trips around BNP only *Notodoris serenae* was found. No compound has been reported from this species yet. Naamidine A (**65**) as well as isonaamidine-A (**66**, Figure 10) have been isolated among 7 other imidazole alkaloids from the family member *Notodoris citrina* (Gulf of Eilat, The Red Sea), feeding on the sponge *Leucetta chagosensis* which also contained the compounds [85], indicating a dietary origin of the metabolites. No bioactivity of the compounds was evaluated at that time. Compound **65**, later isolated from a Fijian *Leucetta*, was evaluated as selective inhibitor of the epidermal growth factor (EGF) and inhibited human tumour xenografts in mice [86]. Additionally, **65** from a Fijian *L. chagosensis* has antitumour activity and promotes caspase-dependent apoptosis in tumour cells [87]. Compound **66**, isolated from the sponge *L. chagosensis* collected at French Polynesia, inhibits strongly the AI-2 channel of *Vibrio harveyi*, a marine pathogen and therefore acts as quorum sensing inhibitor [88]. *Notodoris gardineri* from Philippines contained the imidazole alkaloids **66** and dorimidazole-A (**67**, Figure 10), the latter exhibiting anthelminthic activity [89]. Clathridine (**68**, Figure 10), a sponge derived, cytotoxic imidazole alkaloid was found in samples of *N. gardineri* from the Great Barrier Reef and Papua New Guinea [90,91]. The biosynthetic origin of **68** is presumably the sponge *Clathrina clathrus* [92,93].

#### 3.6.2. Chromodorididae

##### Ceratosoma

**Figure 11:**
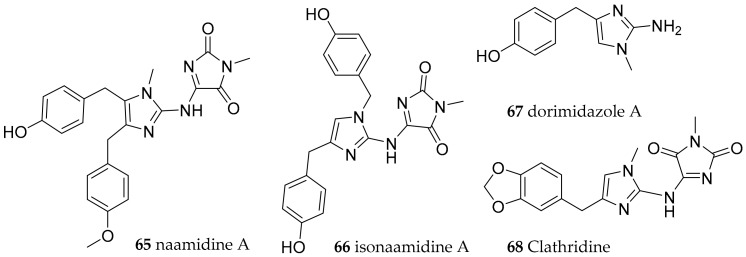
Bioactive metabolites from *Ceratosoma* species.

A *Ceratosoma* sp. 2 has been found at BNP. No bioactive compounds have been reported from this species. However, a *Ceratosoma amoena* from the Great Barrier Reef was reported to contain allolaurinterol (**69**, Figure 11), which was also identified from the red algae *Hymenena variolosa* [94]. Since algae are not food of nudibranchs such as *Ceratosoma*, the authors suggest as explanation feeding of, e.g., *Aplysia* species upon the algae and transfer of the compound to their egg ribbons which are then eaten by the *Ceratosoma* [94]. Compound **69** isolated from *Laurencia obtusa* collected from the Caribbean island of Dominica was described to bear mild antibiotic, antifungal and anti-algal activity [95]. New investigations of **69** isolated from *L. majuscula* and *L. venusta* collected at Tanegashima Island also show good activity against antibiotic resistant bacteria [96]. (−)-Furodysinin (**70**, Figure 11) was the main sesquiterpene metabolite found in *C. trilobatum* and *C. gracillimum*, collected along the South China Sea coast and exhibited significant feeding-deterrent and ichthyotoxic properties. Compound **70** was also detected alongside the feeding deterrent nakafuran-9 (**71**, Figure 11) in *C. gracillimum* specimens from Hainan [97].

##### Chromodoris

Around Bunaken Island we found specimens of *Chromodoris annae*, *C.* cf. *boucheti, C. dianae*, *C. lochi*, *C. strigata* and *C. willani*. From this *Chromodoris* species, only *C. lochi* and *C. annae* have been reported to contain bioactive compounds.

**Figure 12:**
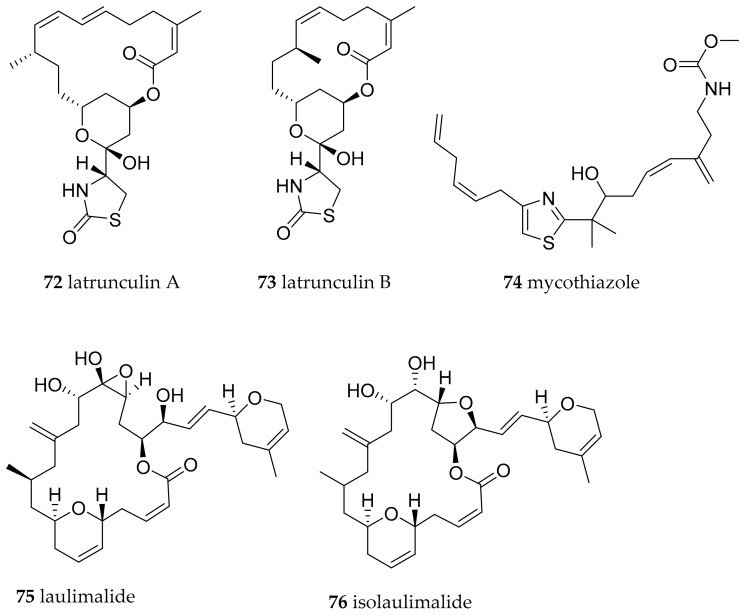
Bioactive metabolites from *Chromodoris lochi.*

The cytotoxic and ichthyotoxic PKS-NRPS derived macrolide latrunculin A (**72**, Figure 12) was the first natural product discovered from *C. lochi* [98]. It was found by an investigation of Fiji specimens and their food source *Spongia (=Cacospongia) mycofijiensis* [98] which also contained these molecules, indicating a dietary origin. Lantrunculin A (**72**) and B (**73**, Figure 12) were first described from the Red Sea sponge *Negombata magnifica* [99] and were also found in other sponges such as *Hyattela* sp. as well as in *C. hamiltoni* from South Africa [100], *C. elisabethina*, *C. magnifica*, *C. kuiteri*, *C. annae* and *C. quadricolor* [101]. The latrunculins bear strong actin binding properties, thus interfering with the cytoskeleton and inhibiting the proliferation of cancer cells. Latrunculin B (**73**) additionally exhibited strong antifungal activity [102].

The PKS-NRPS derived mycothiazole (**74**, Figure 12) was found in *C. lochi* samples from Vanuatu and first reported with anthelmintic and toxic activities [103]. Compound **74** has also been found in sponges such as *C. mycofijiensis* [104] and shows selective cytotoxic activity, inhibits the hypoxia-inducible factor-1 (HIF-1) and suppresses mitochondrial respiration at complex I [105,106]. Analysis of *C. lochi* specimen from Indonesia revealed the presence of the sponge (again *C. mycofijiensis*) derived polyketides laulimalide (syn fijianolide B) (**75**, Figure 12) and isolaulimalide (syn fijianolide A) (**76**, Figure 12) [107]. Both exhibit cytotoxic activity by microtubule-stabilizing action but not at the same binding site as taxanes and are of interest as antitumor agents [108,109].

**Figure 13:**
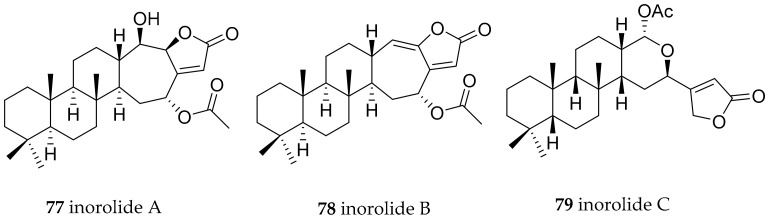
Bioactive metabolites from *Chromodoris aspersa.*

Other species of the genus *Chromodoris* have been investigated and an intriguing diversity of compounds has been found, e.g., the investigation of Japanese *Chromodoris inorata* (accepted as *Chromodoris aspersa*) specimen revealed the presence of the sesquiterpenoids inorolide A (**77**), B (**78**) and C (**79**) (Figure 13) and a mixture of scalaranes. All inorolides were evaluated as being cytotoxic against murine lymphoma L1210 and epidermoid carcinoma KB cell lines [110].

**Figure 14:**
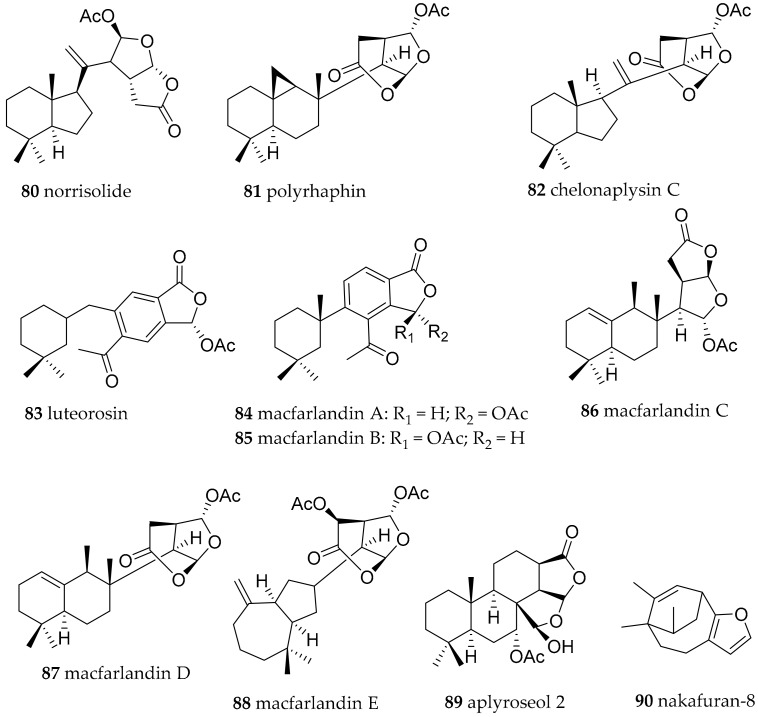
Further bioactive metabolites from *Chromodoris* species.

Analysis of *Chromodoris luteorosea* (accepted as *Felimida luteorosea*) from Spain revealed the presence of the ichthyotoxic diterpenes norrisolide (**80**), polyrhaphin C (**81**) and chelonaplysin C (**82**), luteorosin (**83**), as well as macfarlandin A (**84**) (Figure 14) and the closely related compounds [111]. Compound **80** has been found before in *C. norrisi* (accepted as *Felimida norrisi*) and in several sponges [111]. *Chromodoris macfarlandi* (accepted as *Felimida macfarlandi*, collected in California yielded a greater range of macfarlandines, i.e., macfarlandines A–E (**84**–**88**, Figure 14) [111,112,113]. Compound **88** proved to have unique Golgi-modifying properties that are different from the activities of norrisolide (**80**) [114]. Aplyroseol-2 (**89**, Figure 14), bearing cytotoxic activity was found in *Chromodoris sinensis* (accepted as *Goniobranchus sinensis*) from the South China Sea [97]. It was also found in Australian *Chromodoris reticulata* (accepted as *Goniobranchus reticulatus*) specimen together with other diterpenes [115].

The feeding deterrent nakafuran-9 (**71**, Figure 11) already mentioned from *Ceratosoma gracillimum* was also found in *Hypselodoris maridadilus* (as *Chromodoris maridadilus*) from Hawaii together with its related also feeding deterrent nakafuran-8 (**90**, Figure 14) [116]. Both were also present in the sponge that *Dysidea fragilis, H. maridadilus* feeds on [116].

##### Doriprismatica and Glossodoris

Specimens of *Doriprismatica* (=*Glossodoris*) *stellata* and *Glossodoris* (=*Casella*) *cincta* were found at BNP. None of these species has been chemically investigated so far. However, the closely related *Doriprismatica atromarginata* (as *Glossodoris atromarginata*) has been studied since 1982 [117,118,119], revealing many secondary metabolites of the furanoditerpenoid and scalarane type, and structural variants of these metabolites. These compounds are also found in several sponges and are therefore presumambly diet-derived, e.g., from the sponges *Spongia* sp. (former *Hyatella intestinales*), *Hyrtios erectus* and *Hyrtios* sp. [117,120,121]. Geographic variation between *D. atromarginata* from Sri Lanka and Australia, containing furanoditerpenes, and *D. atromarginata* from India, containing scalarane sesterterpenes has been described as a consequence of sponge prey [122]. These metabolites show various biological activities, such as cytotoxicity, antimicrobial activity, antiviral and antitumor activity, and ichthyotoxicity against *G. affinis* [122,123,124,125,126].

**Figure 15:**
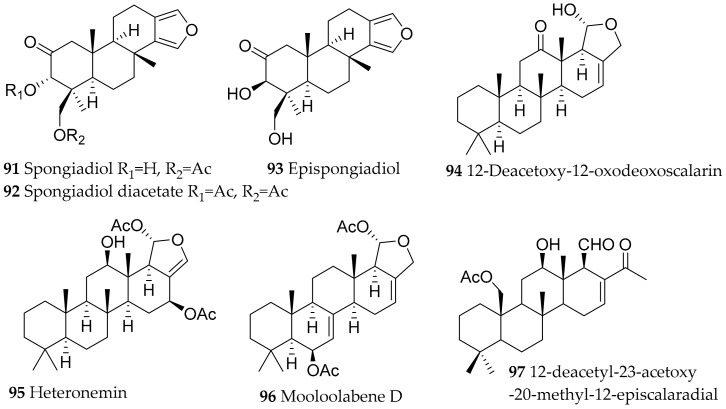
Bioactive metabolites from *Glossodoris* and *Doriprismatica* species.

The most active compounds from *D. atromarginata* were spongiadiol (**91**) [123,127], spongiadiol diacetate (**92**) [128], epispongiadiol (**93**) [123,127,129,130], 12-deacetoxy-12-oxodeoxoscalarin (**94**) [120,124], heteronemin (**95**) [120,125,126,131], and mooloolabene D (**96**) (Figure 15) [122]. In addition to their cytotoxic activity, **91** and **93** showed antiviral, **94** ichthyotoxic and **95** antimicrobial activities.

Further investigation of *Glossodoris dalli* (accepted as *Felimida dalli*), *Glossodoris sedna* (accepted as *Doriprismatica sedna*) [132], *Glossodoris rufomarginata* [124], *Glossodoris pallida*, *Glossodoris vespa* and *Glossodoris averni* (accepted as *Ardeadoris averni*) [119] revealed a series of homoscalarane and scalarane compounds. Of the discovered compounds, 12-deacetyl-23-acetoxy-20-methyl-12-*epi*scalaradial (**97**, Figure 15) showed ichthyotoxicity against *G. affinis* and moderate activity to inhibit mammalian phospholipase A2 [132].

##### Goniobranchus

**Figure 16:**
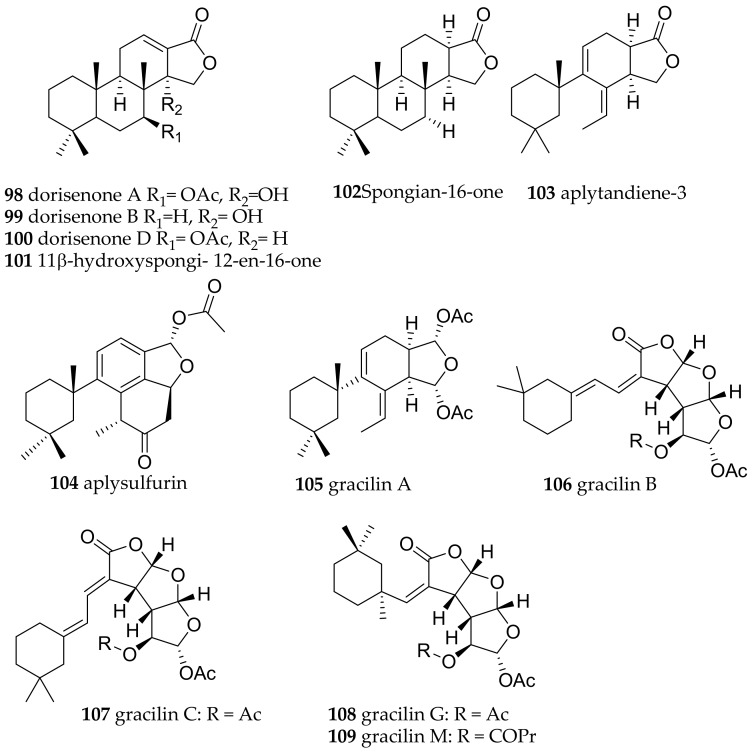
Bioactive metabolites from *Goniobranchus* species.

Specimens of *Goniobranchus geometricus* and *Goniobranchus reticulatus* were found. However, no compounds are reported from these species so far. *Goniobranchus* species are known to contain spongian type cyclic diterpenes. This group of cytotoxic compounds is huge and only structural examples are shown in Figure 16. In *Goniobranchus obsoletus* (former *Chromodoris obsoleta*) for example dorisenones A (**98**), B (**99**), D (**100**), 11β-hydroxyspongi-12-en-16-one (**101**), spongian-16-one (**102**) (Figure 16), together with related compounds were found and exhibited cytotoxicity as low as IC_50_ = 0.2 μg/mL against murine lymphoma LI210 and human epidermoid carcinoma KB cells [133].

Alongside spongian-16-one (**102**), aplytandiene-3 (**103**), aplysulfurin (**104**) and aplyroseol-2 (**89**), gracilins, e.g., gracilin A (**105**), B (**106**), C (**107**), G (**108**), M (**109**) (Figure 16) were isolated from *G.splendidus* from Australia. All metabolites showed cytotoxic activity against Hela S3 cells [134]. Coumpound **106** and **107** and isomers showed activity against a wide panel of human tumor cell lines [135]. Again, gracilins are much better known from sponges such as *Spongionella* sp. than from *Goniobranchus* species and have been investigated as cyclosporine A mimics, as well as as BACE1 and ERK inhibitors. Hence, harbouring potential as antiinflammatory drug candidates, as well as potential in neurodegenerative diseases [136,137].

##### Hypselodoris

*Hypselodoris maculosa* was found near Bunaken. No bioactive compounds are reported from this species, but *Hypselodoris infucata* from Kaneohe Bay, Oahu, Hawaii gave a 3:1 mixture of nakafuran-8 (**90**) and nakafuran-9 (**71**), in the same ratio as found in the prey sponge, *Dysidea fragilis* [116]. Minor metabolites previously isolated from the sponge were, however, not detected in the nudibranch extract in that study. Compounds **71** and **90** are reported as feeding deterrent, but did not show any antimicrobial activity when tested against *E. coli*, *S. aureus*, *P. aeriginosa* and *B. subtilis* in a disk diffusion assay [138]. Results from *Hypselodoris maridadilus* are already outlined above.

#### 3.6.3. Cladobranchia (Families Arminidae, Dotidae, Eubranchidae, Facelinidae, Flabellinidae Proctonotidae)

Sea slugs from 6 families of the Cladobranchia clade have been collected from BNP. The chemical molecules of this clade have recently been reviewed and will not be repeated here [139]. An excellent work on the defense mechanism of *Phyllodesmium* has also recently been published and only the most bioactive compounds are mentioned below [1]. From the Arminidae family, *Dermatobranchus striatus* and two other different *Dermatobranchus* sp. were identified. No bioactive metabolites have been isolated from these species so far. From the family Dotidae, *Doto* sp., and from the family Eubranchidae *Eubranchus* sp. 4 have been found. From the family Facelinidae, *Caloria indica*, *Favorinus japonicus*, *Favorinus mirabilis*, *Favorinus tsuruganus*, *Favorinus* sp., two different species of *Noumeaella* sp. as well as *Phyllodesmium briareum*, *Phyllodesmium poindimiei*, *Facelina rhodopos* and *Pteraeolidia semperi* were identified. Cytotoxic briarane diterpenes such as brianthein W (**110**, P-388—ED_50_ 0.76 μg/mL) and excavatolide C (**111**, P-388—ED_50_ 0.3 μg/mL; KB, A-549, HT-29—ED_50_ 1.9 μg/mL) were isolated from *P. briareum*, and were also identified in its prey *Briareum* sp. (Octocorallia). *Phyllodesmium magnum* yielded several cembrane diterpenes including the cytotoxic 11-episinulariolide acetate (**112**) (P-388 ED_50_—1.2 μg/mL, HT-29 ED_50_ 1.9 μg/mL, HL-60 ED_50_ 0.8 μg/mL).

No bioactive compounds are reported for *Noumeaella*, *Caloria* and *Favorinus* species.

From the family Flabellinidae, several specimens of *Flabellina bicolor*, *F. exoptata*, *F. rubrolineata* were found at BNP. Additionally, at least four unidentified *Flabellina* species were collected.

Of these species, only the secondary metabolite content of *F. exoptata* was investigated [139]. A possible explanation for this low abundance of chemical defense molecules is that this Aeolidoidean molluscs, feeding upon hydrozoans, primarily use cleptocnides as their defense mechanism [140]. Chemical studies of *F. exoptata*, *F. ischitana, F. pedata* and *F. affinis* revealed that they contain homarine (**113**), which is frequently encountered in the Cladobranchia and their food sources. The biological function of **113** is, however, controversial [139,141].

**Figure 17:**
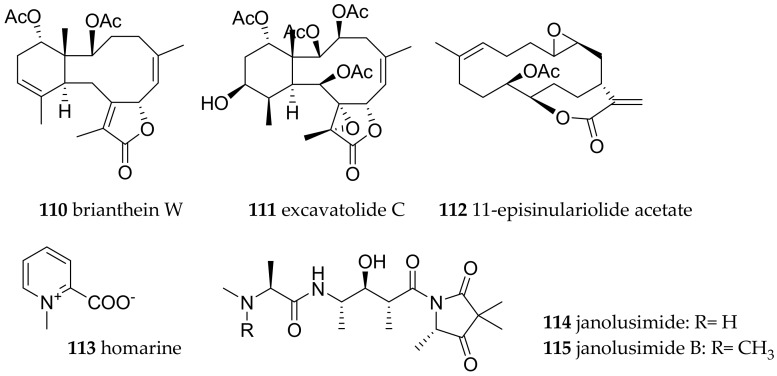
Selected bioactive metabolites from *Cladobranchia* species.

From the family Proctonotidae, *Janolus* sp. (sp.11 Gosliner) was collected at BNP. The only literature of bioactive compounds from the nudibranch family Proctonotidae comes from a Mediterranean *Janolus cristatus* containing the toxic tripeptide janolusimide (**114**) (LD 5mg/kg; i.p. for mice) [139,142]. Until recently, no such tripeptides had been known from the food source of *Janolus*, but very recently, the *N*-methyl analogue janolusimide B (**115**, Figure 17) has been isolated from a New Zealand bryozoan *Bugula flabellata* [143].

#### 3.6.4. Dendrodorididae

At BNP, specimens of *Dendrodoris* cf. *fumata* and *Dendrodoris nigra* were found. Extracts of the eggmasses of *D. fumata* showed antibacterial activity against *E. coli, Staphylococcus aureus* and *Pseudomonas aeruginosa*, indicating the use of chemical defence to protect early stage embryos against bacterial infection [144]. Chemical studies of *D. nigra*, *D. carbunculosa* as well as other Dendrodorids, have revealed the presence of many de novo synthesized sesquiterpenes of the drimane series, which can be considered a chemical marker of these nudibranchs [118,145,146,147,148].

**Figure 18:**
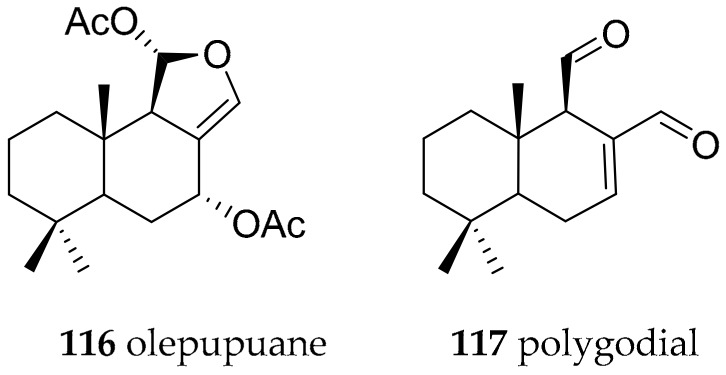
Bioactive metabolites from *Dendrodoris* species.

Olepupuane (**116**) and polygodial (**117**) (Figure 18), a previously known feeding deterrent plant metabolite [149], have been isolated from different dendrodorid nudibranchs including *Dendrodoris limbata* [147]. Compound **117** exhibits antifeedant properties against marine and freshwater fish [150] and shows antifungal activity against *Saccharomyces cerevisiae* IFO 0203 and *Hansenula anomala* IFO 0136 [151]. However, **117** may not be originally present in the nudibranchs in a free state, due to its marked chemical reactivity towards primary amino groups [152], which could cause extensive damage to the nudibranch proteins. It was concluded that **116**, de novo synthesized by dendrodorid slugs, is the easily stored, masked form of polygodial, which is transformed into the potent antifeedant when in contact with predators [152].

#### 3.6.5. Discodorididae

**Figure 19:**
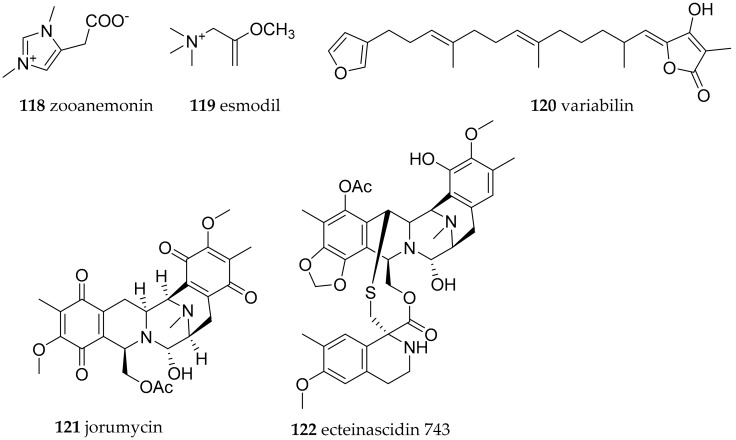
Bioactive metabolites from *Discodoris* species, ecteinascidin 743.

*Taringa halgerda*, *Halgerda carlsoni* and *H. tessellata* were collected at BNP. For none of these species bioactive natural products have been reported so far. An investigation of 5 *Halgerda* species from Australia and Japan, i.e., *H. aurantiomaculata*, *H. gunessi*, *H. rubicunda*, *H. theobroma* and *H. willeyi* showed that only *H. aurantiomaculata* contained tryptophane derivatives. Zooanemonin (**118**, Figure 19), was previously isolated from different marine sponges and the sea anemone *Anemonia sulcate* and was reported as antibacterial, whereas esmodil (**119**, Figure 19) first described as a synthetic compound, but also reported from the sponge *Raspailia* sp., has been described as a muscarinic agonist [153].

*Paradoris indecora* from Spain and Italy, former *Discodoris indecora*, has been described to contain furano sesterterpenes including variabilin (**120**, Figure 19) [154]. The latter was not toxic to *G. affinis* at 10 ppm, but active at a concentration of 300 µg/cm^2^ in an antifeeding assay using fresh water and marine fishes.

The most bioactive compound found in the family Discodorididae was first isolated from a *Jorunna funebris* collected of Mandapam (India). Jorumycin (**121**) is an isoquinolin alkaloid with antitumor activity of IC_50_ = 12.5 ng/mL against cancer cell lines P388, A549, HT29 and MEL28 and has a saframycin-like structure similar to one of the most active marine–derived antitumor agents ecteinascidin 743 (**122**, Figure 19) isolated from the tunicate *Ecteinascidia turbinata* and an approved drug [155,156,157]. The development of ecteinascidin 743 as a drug shows clearly that overcoming the supply problem very often relies on bacteria, e.g., *Pseudomonas fluorescens* to produce a suitable precursor for synthesis of the final product [158].

#### 3.6.6. Hexabranchidae

**Figure 20:**
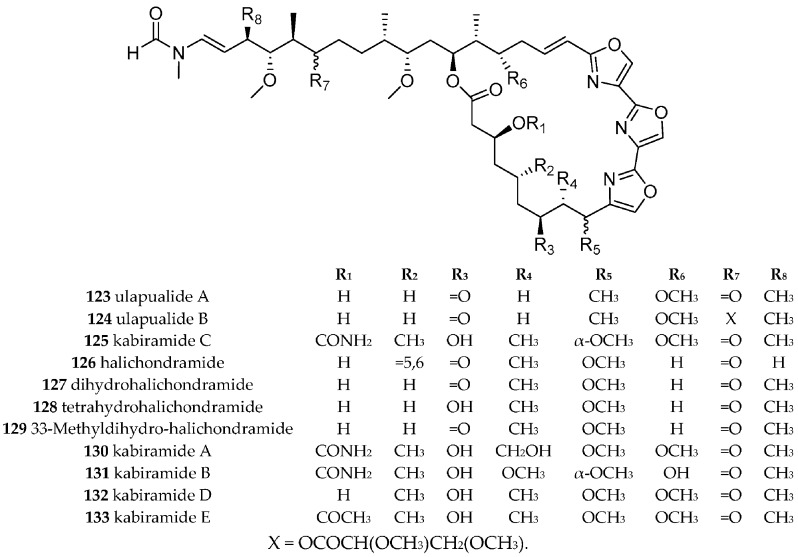
Kabiramides and halichodramides isolated from *Hexabranchus sanguineus* or its egg mass and from various sponges.

The family Hexabranchidae consist only of one genus consisting of two species. The egg mass of one of them, i.e., *Hexabranchus sanguineus* was collected at BNP. The first trisoxazole macrolides, i.e., ulapualide A (**123**) and B (**124**) (Figure 20) were isolated from egg masses of a Hawaiian *H. sanguineus*, [159] and from an unidentified nudibranch egg mass (kabiramide C, **125**) from Kabira Bay, Ishigaki-jima Island, Japan [160]. The ulapualides were reported to inhibit proliferation of L1210 leukemia cells (IC_50_ 0.01–0.03 µg/mL) and the growth of *Candida albicans*, while **125** inhibited various fungi. Very recently, more ulapualides have been isolated and found to be less cytotoxic than **123** and **124 [161]**. Halichondramides **126**–**129** and kabiramide A–E (**125** for C and **130**–**133** Figure 20), isolated from *H. sanguineus* and the sponge *Halichondria sp*., were antifungal, cytotoxic and deterred *Thalassoma lunare* [162,163,164].

The trisoxazole macrolides bind to actin and can be regarded as a small molecule biomimetic of the gelsolin actin-binding proteins with drug lead potential—at least of the tail part of the molecule [165,166,167,168].

*H. sanguineus* seems to be the only nudibranch source of trisoxazole macrolides, but more than 30 highly similar trisoxazole macrolide have been isolated from different sponges, e.g., from the genera *Halichondria, Mycale, Jaspis* and *Pachastrissa* [160,165,166,167,169]. Some of them are reported to be a food source of *H. sanguineus*, and given the choice, *H. sanguineus* fed only on trisoxazole containing *Halichondria* and not on other sponges [162]. The intriguing structure of the trisoxazole macrolides is presumably of hybrid polyketide—non-ribosomal peptide biosynthesis. The producer is most likely an as yet unidentified microorganism, suggested by comparison with other actin binding macrolides such as luminaolide (first isolated from a red algae with its biosynthesis genes discovered in a cyanobacteria) [170]. Various total syntheses of marine macrolides including the trisoxazole macrolides are reported and reviewed by Yeung and Paterson [171] whereas the focus for chemical synthesis is nowadays on the bioactive tail part [168,172].

#### 3.6.7. Phyllidiidae

In the family Phyllidiidae, more than 49 species are found, which live in most parts of the Indo-Pacific. Intensive phylogenetic analysis to assist identification has been conducted using morphological and molecular data of 99 specimens (16 species) from Indonesian waters (West Papua, Gam, Kri, Mansuar, Batanta, Halmahera, Ternate) [173]. Species like *Phyllidiella pustulosa* yielded several subclades whereas molecular marker confirmed morphologically variable species like *P. elegans* and *P. varicosa* to be correct concerning the species boundaries [173]. Extensive chemical investigations of some species have been reported and focus on isocyanide substituted terpenes [174]. For sponges it was shown, that such an isonitrile function originates from inorganic cyanide. The terpenes possess various carbon skeletons, e.g., amorphane, pupukeanane, bisabolane. They are most probably derived from the sponge diet of the slugs and serve as defense chemicals against predators. Isocyanides also have antifouling activity, since they inhibited the settlement of barnacles [175]. From field trips in the BNP *Phyllidia coelestis*, *Phyllidia elegans*, *Phyllidia ocellata*, *Phyllidia varicosa*, *Phyllidiella annulata*, *Phyllidiella pustulosa*, *Phyllidiella* cf. *lizae*, *Phyllidiella striata*, *Phyllidiopsis pipeki* and *Phyllidiopsis sphingis* were found.

*Phyllidia elegans, Phyllidiella annulata, Phyllidiella* cf. *lizae* as well as *Phyllidiopsis* species have not been investigated yet concerning their secondary metabolites. The ethyl acetate extract of *P. elegans* from Guam was feeding deterrent towards reef fish, but no secondary metabolites were reported [174].

**Figure 21:**
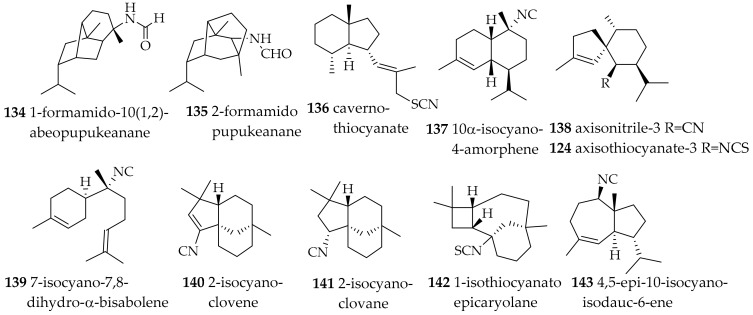
Bioactive sesquiterpenes from *Phyllidia* species.

*Phyllidia coelestis* from Koh-Ha Islets, Krabi Province, Thailand, was recently reported to contain 1-formamido-10-(1,2)-abeopupukeanane (**134**, Figure 21), an unprecedented sesquiterpene with a rearranged and brigded carbon skeleton. Its structure was deduced based on extensive NMR data, but also X-ray analysis. It is structurally similar to 2-formamidopupukeanane (**135**), which was also reported in this study from *P. coelestis*. The formamide moiety in these compounds is most probably derived from an original isonitrile function [176]. Compounds **134** and **135** have cytotoxic properties towards cancer cells in the range of 0.05–10 µM [177]. *P. coelestis* was also investigated for its lipid classes and fatty acid composition revealing unusual fatty acids, e.g., very long chain fatty acids [178].

Two reports target the secondary metabolite chemistry of *P. ocellata*. The sponge *Acanthella* cf. *cavernosa* from Hachijo-jima Island in Japan and *P. ocellata*, which most likely preyed upon this sponge, were investigated. Most secondary metabolites of the sponge were also found in the slug, i.e., cavernothiocyanate (136), 10α-isocyano-4-amorphene (137), axisonitrile-3 (**138**), and 7-isocyano-7,8-dihydro-α-bisabolene (**139**, Figure 21) [179]. *P. ocellata* from Australian waters yielded sesquiterpenes also with isonitrile and isothiocyanate groups, however with different carbon skeletons as compared to the Japanese samples, e.g., 2-isocyanoclovene (**140**) and its dihydro analogue 2-isocyanoclovane (**141**), 1-isothiocyanatoepicaryolane (**142**) and 4,5-epi-10-isocyanoisodauc-6-ene (**143**, Figure 21). In vitro antimalarial activity tested for **140**–**143** was traced back to the isocyano functionality in the metabolites with IC_50_ values of 0.26−0.30 μM for **140**, **141** and **143** vs. >10 μM for **142** [180].

*P. varicosa* seems to display much variability concerning its coloration, and *P. varicosa* and *P. arabica* are regarded as synonyms. In part, this taxonomic uncertainty can be traced back to alterations during preservation with regard to colour [181].

**Figure 22:**
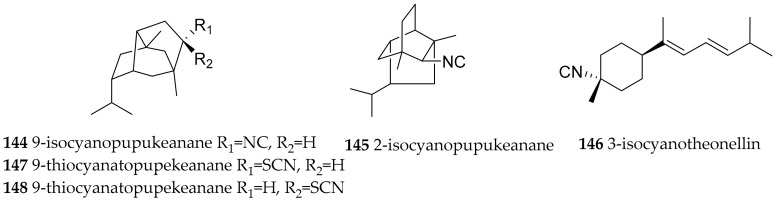
Bioactive sesquiterpenes from *Phyllidia varicosa*.

*P. varicosa* extracts (collected from Palau) deterred feeding by fish [174], and early observations reported that secretions from *P. varicosa* were lethal to fish and crustaceans. The toxic principle was already described in 1975 as a tricyclic sesquiterpene isocyanide, which at the time had a novel rearranged isoprenoid skeleton. According to the location of collection, i.e., Pupukea (Oahu, Hawaii) the compound was called 9-isocyanopupukeanane (**144**). The compound was also detected in the sponge *Ciocalypta* sp. (ex. *Hymeniacidon* sp.) on which *P. varicosa* was feeding [182]. Later, also 2-isocyanopupukeanane (**145**, Figure 22) was identified in *P. varicosa* from the same location [183]. In Japanese waters *Phyllidiella rosans* (former name *Phyllidia bourguini*) also yielded **144** [184]. From a Sri Lankan sample of *P. varicosa* the bisabolene type 3-isocyanotheonellin (**146**, Figure 22) with antifouling properties was obtained [185].

*P. varicosa* and the sponge *Axinyssa* cf. *aculeata* on which it preyed were obtained from reefs of Pramuka Island, Thousand Islands National Park, Indonesia. The nudibranch is one of the most abundant slugs in this National Park of Indonesia. Sponge and nudibranch contained epimeric 9-thiocyanatopupekeanane sesquiterpenes (**147**, **148**, Figure 22) which were determined to be toxic to brine shrimp (LC_50_ 5 ppm) together with **144 [186]**.

*Phyllidiella pustulosa* is quite well investigated today. In a biosynthetic study addressing the origin of the isocyanide and isothiocyanate functionality in axisonitrile-3 (**138**) and axisothiocyanate-3 (**149**) (Figure 21), metabolites of the sponge *Acanthella cavernosa*, also *P. pustulosa*, which fed on this sponge, was investigated. Using ^14^C-labelled potassium cyanide the sponge metabolites were ^14^C enriched and subsequently, the labeled terpenes could be found in the nudibranch [176].

**Figure 23:**
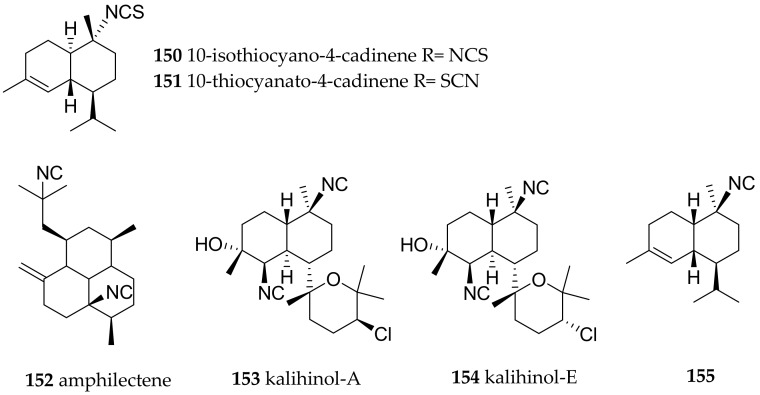
Bioactive sesquiterpenes from *Phyllidiella pustulosa*.

*P. pustulosa* from Nananu-I-Ra reef, Fiji, presumably feeds on the sponge *Phakellia carduus* as judged from the very similar secondary metabolites present in the both samples. Apart from **138** (Figure 21), 10-isothiocyano-4-cadinene (**150**, Figure 23) with moderate antiplasmodial activity was reported [187]. This structure, however was questioned, based on a synthetic approach, and it was suspected that Wright had instead isolated 10-thiocyanato-4-cadinene (**151**, Figure 23), i.e., with a thiocyanate in place of the isothiocyanate [188]. The latter study demonstrated that the structure determination of such compounds is a major challenge.

In Chinese samples (Hainan Island, South China Sea) of *P. pustulosa* di- and sesquiterpenes were found. The diterpenes amphilectene (**152**), kalihinol-A (**153**), kalihinol-E (**154**) (Figure 23) were before reported from sponges, and the sesquiterpene named ent-stylotelline was the enantiomer of the sponge metabolite stylotellin. Compound **146** (Figure 22) was also found here, as above described for *P. varicosa*. The number of different and intriguing carbon frame works in this sample is astonishing. Feeding-deterrence tests against goldfish (*Carassius auratus*) revealed **152**–**154** to be active at 50 µg/cm^2^ [175].

From the coasts of Vietnam *P. pustulosa* yielded several sesquiterpenes, including **144**, its C9-epi isomer and several sterols and ketosteroids. Based on the terpenes obtained the authors concluded that the mollusk feeds on sponges of the genera *Acanthella, Halichondria, Axinella* and *Axinyssa* [189]. *P. pustulosa* collected at Kin Bay, Okinawa, yielded **138**, substituted axinisothiocyanate K derivative, and a new molecule (**155**) (Figure 23) with an isocyano group. The compounds were found to be moderately cytotoxic [190].

#### 3.6.8. Polyceridae

From the family Polyceridae we collected Nembrotha cristata, Nembrotha kubaryana, Kaloplocamus dokte, Polycera japonica and Polycera risbeci during the field trips at BNP. No compounds are reported for the genus Kaloplocamus yet. The genus Tambja in the subfamily Nembrothinae gave the name to the tambjamines, and field and lab observations with species collected at the West coast of America clearly established that T. abdere and T. eliora favored the tambjamine containing bryozoan Sessibugula translucens rather than, e.g., Bugula neritida as a food source. In turn, they were eaten by Roboastra tigris, also a sea slug of the Polyceridae family [191]. Nembrotha species collected in Micronesia have also been found to contain the presumably diet derived tambjamines A (**156**), C (**157**), E (**158**), F (**159**) the tambjamine aldehyde (**160**) and the blue tetrapyrrol (**161**) which were also found in the slugs prey the ascidian Atapozoa sp. [138,192]. From Nembrotha cristata from Ant Atoll the ratio of **156**:**157**:**158**:**159**:**160** was 39:39:5.5:11:5.5. From Nembrotha kubaryana from Sumilon Island, Philippines the ratio of **157**:**158**:**161** was 30.8:30.8:38.4 and from Nembrotha sp. from Apo Islands, Philippines **157**:**158**:**159**:**160** was 11.8:47:5.9:35.3. The crude extracts, mixtures of tambjamines, **157**, **159** and **161** were all significant feeding deterrents at or below natural concentrations. The tambjamines **156** and **158** were not deterrent when tested alone at natural concentrations [192]. Compound **161** was found to be a potent antimicrobial agent; active against Bacillus subtilus at 5 μg/disc [138].

No bioactive metabolites are reported for the two *Polycera* species, but *Polycera atra* MacFarland, 1905, which feeds on the byozoan *Bugula neritina*, contains bryostatins in its body and egg masses [193,194,195].

**Figure 24:**
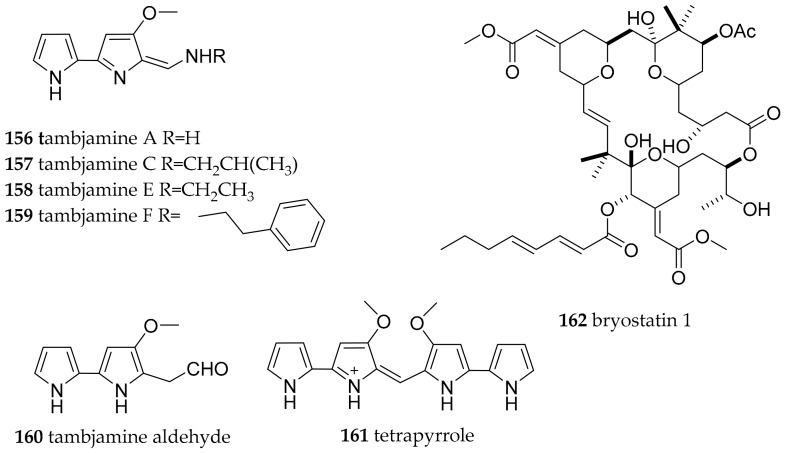
Biologically active natural products isolated from sea slugs of the family Polyceridae.

Bryostatins are a group of 20 polyketide macrolides with bryostatin 1 (**162**, Figure 24) investigated in over 20 clinical trials for treatment of cancer and Alzheimers disease. The true producer is an uncultured symbiotic bacterium *Candidatus endobugula sertula* and the biosynthesis genes of this polyketide have been discovered [196].

## 4. Discussion and Conclusions

It can be seen that many compounds isolated from heterobranchs are most likely produced by invertebrate associated bacteria or cyanobacteria and Ciavatta concludes: “Noteworthy, the “mollusk-derived” metabolites that entered or that are in clinical trials are actually produced by microbes” [197]. However, the sea slugs themselves represent a great opportunity to screen for natural compounds with biological activity. It seems that evolution directed the sequestration of metabolites from the food source in a way that compounds providing a benefit for the sea slug, e.g., protection due to the fact that the metabolite has repellent activity against predators, are enriched. As a consequence, it is much easier to detect compounds with promising biological activities from the sea slugs, even though they do not represent the primary producer. Further research is needed to reveal the true producer of a bioactive compound, which is expected to be often a microorganism associated with the prey. Hence, the isolation of compound-producing microorganisms might be an approach to solve the supply issue [198]. Even if the true producer is not yet culturable, a metagenomic toolbox is available to discover the biosynthetic gene cluster corresponding to the natural product of interest and using it for heterologous expression approaches [199]. Securing the supply will facilitate comprehensive analysis and testing in the lab, which is a precondition for translational research, once natural products enter drug development phase.

From the huge biodiversity of sea slugs found around Bunaken Island, only a minority of species (14 out of 73) has been investigated chemically so far with only three from Indonesia (Table 1 and Table 2). Here, *Pleurobranchus forskalii* and *Chromodoris lochi* show a diversity of highly bioactive chemically not related compounds, which are dietary derived and some are presumably of microbial origin. Looking then at the family level of the Chromodorididae (a very specious family) with 12 species found at BNP, the literature shows 41 bioactive compounds reported from 26 species worldwide with terpenoids dominating (see Table 2), but only *C. lochi* is included from the species collected at BNP. Because the sea slugs can usually shift to another food source if necessary, it is hard to determine a chemical marker for Chromodorididae. However, since this family is a proven rich source of bioactive compounds and many species are still under-investigated, it can be concluded that the chance to detect further interesting compounds is high.

For upcoming screening approaches, a valid dereplication strategy must be employed to identify novel natural products and to prevent detection of already known compounds over and over again, e.g., the online platform Global Natural Products Social Molecular Networking [200], which uses MS/MS data to compare molecular features. Even though, a correlation between taxonomic distance and the production of distinct secondary metabolite families can be expected, as it was recently statistically verified for myxobacterial species [201], also closely related species might harbour new metabolites in challenging habitats, e.g., reefs.

In conclusion, the presented review of bioactive compounds obtained from heterobranchs shows the enormous potential of sea slugs if sufficient material can be obtained, or the food chain can be used to identify a compound. The structural diversity stretches from very diverse terpenes, e.g., the macfarlandines A–E (**84**–**88**) and briaranes (**110**–**111**) over peptides, e.g., kulolide (**31**), polyketides e.g., laulimalide (**75**) to complex imidazole alkaloids, e.g., **65**, **66** and the highly cytotoxic jorumycin (**121**), as well as structures which should be biosynthesized by mixed biosynthetic pathways like the highly cytotoxic kabiramides (**125**, **130**–**133**). Considering coral reef environments, it was shown that three genera of stony corals had distinct patterns of molecular relatedness, despite their high degree of taxonomic relatedness [202]. Further, it was indicated that even between individuals, different metabolomes exist, suggesting that every coral reef holobiont is a potential source of novel chemical diversity [202]. Hence, a biodiversity hot spot like BNP is a promising habitat for the detection of novel natural products and it underlines the need for biodiversity conservation to keep the reservoir of novel compounds beneficial for human wellbeing. In addition, the biodiversity hotspot of Indonesia is also a great place to study bio systems including food chain and defence mechanisms of sea slugs.

## Figures and Tables

**Table 1:** Species diversity collected at Bunaken National Park (BNP) grouped by phylogenetic relatedness, localities of their collection, depth and abundance and indication of known chemistry.

Clade		Family	Species	Locality	Depth and Abundance ^a^	Bioactive Compounds
Anaspidae (sea hares)		Aplysiidae Lamarck, 1809	Stylocheilus striatus (Quoy and Gaimard, 1832)	Bunaken	10 m; rare	see text and Table 2
Sacoglossa		Caliphyllidae Tiberi, 1881	Cyerce sp. 4 (cf. Cyerce bourbonica Yonow, 2012)	Bunaken	3–10 m; uncommon	nothing published
			Cyerce sp. 2	Bunaken	3–7 m; uncommon	nothing published
		Oxynoidae Stoliczka, 1868 (1847)	Lobiger sp. 1	Bunaken	7 m; rare	nothing published
			Lobiger viridis (Pease, 1863)	Bunaken	8 m; rare	nothing published
		Plakobranchidae Gray, 1840	Elysia asbecki Wägele, Stemmer, (Burghardt and Händeler, 2010)	Bunaken, Manado Tua, Siladen, Tiwoho	4–15 m; uncommon	nothing published
			3 undescribed Elysia species	Bunaken, Siladen	5–9 m; rare	nothing published
			Thuridilla albopustulosa (Gosliner, 1995)	Bunaken	6 m; rare	nothing published
			Thuridilla flavomaculata (Gosliner, 1995)	Bunaken	2–7 m; rare	nothing published
			Thuridilla gracilis (Risbec, 1928)	Bunaken, Siladen	3–8 m; uncommon	nothing published
			Thuridilla lineolata (Bergh, 1905)	Bunaken, Siladen, Tiwoho	1–9 m; abundant	nothing published
Cephalaspidea		Aglajidae Pilsbry, 1895 (1847)	unidentified specimen	Tiwoho	5 m; rare	nothing published
			Chelidonura amoena (Bergh, 1905)	Bunaken	1 m; rare	nothing published
			Chelidonura hirundinina (Quoy and Gaimard, 1833)	Bunaken, Manado Tua	5 m; rare	nothing published
			Odontoglaja guamensis (Rudman, 1978)	Bunaken, Manado Tua, Tiwoho	3–19 m; uncommon	nothing published
		Diaphanidae Odhner, 1914 (1857)	Colpodaspis thompsoni (G. H. Brown, 1979)	Bunaken, Manado Tua, Siladen, Tiwoho	4–11 m; uncommon	nothing published
		Gastropteridae Swainson, 1840	Sagaminopteron psychedelicum (Carlson and Hoff, 1974)	Bunaken, Manado Tua	4–15 m; uncommon	see text and Table 2
			Siphopteron brunneomarginatum (Carlson and Hoff, 1974)	Bunaken, Manado Tua, Siladen	4–10 m; uncommon	nothing published
			Siphopteron cf. ladrones (Carlson and Hoff, 1974)	Bunaken	5 m; rare	nothing published
			Siphopteron nigromarginatum (Gosliner, 1989)	Manado Tua	5 m; rare	nothing published
			Siphopteron spec.	Bunaken	4–5 m; rare	nothing published
			Siphopteron tigrinum (Gosliner, 1989)	Bunaken	5–6 m; rare	nothing published
		Haminoeidae Pilsbry, 1895	2 undescribed Haminoea species	Bunaken, Manado Tua, Siladen	3–13 m; rare	nothing published
Pleurobranchomorpha		Pleurobranchidae Gray, 1827	Pleurobranchus forskalii (Rüppell and Leuckart, 1828)	Bunaken, Siladen	4–8 m; common	see text and text and Table 2
Nudibranchia	Anthobranchia	Aegiridae P. Fischer, 1883	Notodoris serenae (Gosliner and Behrens, 1997)	Manado Tua	13 m; rare	nothing published
		Chromodorididae Bergh, 1891	Ceratosoma sp. 2	Bunaken	5–8 m; rare	nothing published
			Chromodoris annae (Bergh, 1877)	Bunaken, Manado Tua, Siladen, Tiwoho	4–23 m; abundant	nothing published
			Chromodoris cf. boucheti (Rudman, 1982)	Siladen	8 m; rare	nothing published
			Chromodoris dianae (Gosliner and Behrens, 1998)	Bunaken, Manado Tua, Siladen, Tiwoho	4–21 m; abundant	nothing published
			Chromodoris lochi (Rudman, 1982)	Bunaken, Manado Tua, Siladen	5–17 m; common	see text and Table 2
			Chromodoris strigata (Rudman, 1982)	Siladen	11 m; rare	nothing published
			Chromodoris willani (Rudman, 1982)	Bunaken, Manado Tua, Siladen	7–21 m; common	nothing published
			Doriprismatica (=Glossodoris) stellata (Rudman, 1986)	Bunaken	4–21 m; uncommon	nothing published
			Glossodoris (=Casella) cincta (Bergh, 1888)	Tiwoho	6 m; rare	nothing published
			Goniobranchus geometricus (Risbec, 1928)	Bunaken, Tiwoho	4–8 m; rare	nothing published
			Goniobranchus reticulatus (Quoy and Gaimard, 1832)	Manado Tua	15 m; rare	nothing published
			Hypselodoris maculosa (Pease, 1871)	Bunaken, Tiwoho	4–6 m; rare	nothing published
		Dendrodorididae O’Donoghue, 1924 (1864)	Dendrodoris albobrunnea (Allan, 1933)	Bunaken	4 m; rare	nothing published
			Dendrodoris nigra (Stimpson, 1855)	Bunaken	4 m; rare	see text and Table 2
		Discodorididae Bergh, 1891	Taringa halgerda (Gosliner and Behrens, 1998)	Bunaken, Tiwoho	6 m; rare	nothing published
			Halgerda carlsoni (Rudman, 1978)	Manado Tua	5 m; rare	nothing published
			Halgerda tessellata (Bergh, 1880)	Siladen	5 m; rare	nothing published
			Rostanga sp. 4	Manado Tua	13m; rare	nothing published
		Hexabranchidae Bergh, 1891	Hexabranchus sanguineus (Rüppell and Leuckart, 1830)	Bunaken	2 m; rare	see text and Table 2
		Goniodorididae H. Adams and A. Adams, 1854	Trapania euryeia (Gosliner and Fahay, 2008)	Bunaken	6 m; rare	nothing published
		Gymnodorididae Odhner, 1941	Gymnodoris sp.	Bunaken, Manado Tua	5–7 m; rare	nothing published
		Phyllidiidae Rafinesque, 1814	Phyllidia coelestis (Bergh, 1905)	Bunaken, Manado Tua, Tiwoho	2–15 m	see text and Table 2
			Phyllidia elegans (Bergh, 1869)	Bunaken, Siladen	2–19 m; uncommon	nothing published
			Phyllidia ocellata (Cuvier, 1804)	Tiwoho	5 m; rare	see text and Table 2
			Phyllidia varicosa (Lamarck, 1801)	Bunaken	4–21 m; uncommon	see text and Table 2
			Phyllidiella annulata (Gray, 1853)	Bunaken, Manado Tua	11–13 m; rare	nothing published
			Phyllidiella pustulosa (Cuvier, 1804)	Bunaken, Manado Tua, Siladen, Tiwoho	5–19	see text and Table 2
			Phyllidiella striata (Bergh, 1889)	Bunaken	15 m; rare	nothing published
			Phyllidiopsis pipeki (Brunckhorst, 1993)	Bunaken	14–15 m; rare	nothing published
			Phyllidiopsis sphingis (Brunckhorst, 1993)	Manado Tua	19 m; rare	nothing published
		Polyceridae Alder and Hancock, 1845	Nembrotha cristata (Bergh, 1877)	Bunaken, Siladen	4–15 m; rare	see text and Table 2
			Nembrotha kubaryana (Bergh, 1877)	Tiwoho	6 m; rare	see text and Table 2
			Kaloplocamus dokte (Vallès and Gosliner, 2006)	Bunaken	7 m; rare	nothing published
			Polycera japonica (Baba, 1949)	Bunaken	7–8 m; rare	nothing published
			Polycera risbeci (Odhner, 1941)	Bunaken	7–8 m; rare	nothing published
Nudibranchia	Subclade Cladobranchia	Arminidae Iredale and O’Donoghue, 1923 (1841)	Dermatobranchus fasciatus (Gosliner and Fahey, 2011)	Bunaken	7 m; rare	nothing published
			Dermatobranchus striatus (van Hasselt, 1824)	Manado Tua	7 m; rare	nothing published
		Eubranchidae Odhner, 1934	Eubranchus sp. 4	Bunaken	18 m; rare	nothing published
		Facelinidae Bergh, 1889	Caloria indica (Bergh, 1896)	Bunaken, Manado Tua, Siladen, Tiwoho	3–6 m; uncommon	nothing published
			Favorinus japonicus (Baba, 1949)	Bunaken, Siladen	5–10 m; uncommon	nothing published
			Favorinus mirabilis (Baba, 1955)	Bunaken	23 m; rare	nothing published
			Favorinus tsuruganus (Baba and Abe, 1964)	Bunaken	6–23 m; uncommon	nothing published
			Noumeaella sp. No. 1–2	Bunaken	4–12 m; uncommon	nothing published
			Phyllodesmium briareum (Bergh, 1896)	Bunaken, Tiwoho	2–7 m; abundant	see text and Table 2
			Phyllodesmium poindimiei (Risbec, 1928)	Bunaken	17 m; rare	nothing published
			Facelina rhodopos (Yonow, 2000)	Manado Tua	15 m; rare	nothing published
			Pteraeolidia semperi (Bergh, 1870)	Bunaken, Manado Tua, Siladen, Tiwoho	4–15 m; common	nothing published
		Flabellinidae Bergh, 1889	Flabellina bicolor (Kelaart, 1858)	Siladen, Tiwoho	3–6 m; rare	nothing published
			Flabellina exoptata (Gosliner and Willan, 1991)	Bunaken, Siladen	5–8 m; uncommon	see text and Table 2
			Flabellina rubrolineata (O’Donoghue, 1929)	Bunaken	6 m; rare	nothing published
		Proctonotidae Gray, 1853	Janolus sp. (sp. 11 Gosliner et al., 2015: 308)	Bunaken	7 m; rare	nothing published

^a^ Abundance is classified as: abundant: >40, common 20–39, uncommon 4–19, rare 1–3 specimens.

**Table 2:** Bioactive natural products and their putative origin identified in Heterobranch families that have representatives at BNP.

Clade	Family	Species	Location	Chemistry	(Presumable) Origin of Compound	References
Anaspidae (sea hares)	Aplysiidae Lamarck, 1809	*Dolabella auricularia* (Lightfoot, 1786)	Western Indian Ocean (Mauritius)	dolastatin 10 (**1**)	dietary (cyanobacteria)	[21]
		*Stylocheilus striatus* (Quoy and Gaimard, 1832)	Australia (feeding study)	debromoaplysiatoxin (**3**), lyngbyatoxin A (**6**),	dietary (cyanobacteria *Lyngbya majuscula*)	[22,23,24,25,26,32,37]
		*Stylocheilus longicauda* (Quoy and Gaimard, 1825)	not given, presumably Hawaii	aplysiatoxin (**2**), debromoaplysiatoxin (**3**)	dietary (cyanobacteria)	[22,23,24,25,26]
		*Stylocheilus longicauda* (Quoy and Gaimard, 1825)	Black Point, Oahu, Hawaii	makalika ester (**4**), makalikone ester (**5**), lyngbyatoxin A acetate (**7**), malyngamide O (**10**)	dietary (cyanobacteria)	[27,28,29,30,31,35]
		*Stylocheilus longicauda* (Quoy and Gaimard, 1825)	not given, presumably Hawaii	kulolide-1 (**31**)	dietary (cyanobacteria)	[63]
		*Bursatella leachii* (Blainville, 1817)	Australia (feeding study)	lyngbyatoxin A (**6**)	dietary (cyanobacteria)	[37]
Sacoglossa	Caliphyllidae Tiberi, 1881	*Cyerce cristallina* (Trinchese, 1881)	Mediterranean Sea	cyercene A **11**) and B (**12**) and 1–5 (**13**–**17**)	de novo	[2,38]
		*Cyerce nigricans* (Pease, 1866)	Lizard Island (Australia)	chlorodesmin (**18**)	diatery (green alga *Chlorodesmis fastigiata)*	[39,40]
	Oxynoidae Stoliczka, 1868 (1847)	*Lobiger serradifalci* (Calcara, 1840)	Capo Miseno (Bay of Naples, Italy)	oxytoxin-1 (**20**)	modified from dietary caulerpynene (**19**) from green alga *Caulerpa prolifera*	[41,42,43]
		*Oxynoe olivacea* (Rafinesque, 1814)	Murcia (SE Spain)	oxytoxin-1 (**20**), oxytoxin-2 (**21**)	modified from dietary caulerpynene (**19**) from green alga *Caulerpa prolifera*	[41,42,43]
		*Oxynoe olivacea* (Rafinesque, 1814)	Bay of Naples, Italy	oxytoxin-1 (**20**), oxytoxin-2 (**21**)	modified from dietary caulerpynene (**19**) from green alga *Caulerpa prolifera*	[41,42,43]
	Plakobranchidae Gray, 1840	*Elysia chlorotica* (Gould, 1870)	Bay of Fundy, Canada	elysione (**22**)	de novo	[3,51]
		*Elysia viridis* (Montagu, 1804)	Fusaro Lake, Bay of Naples, Italy	elysione (**22**)	de novo	[3,51]
		*Elysia translucens* (Pruvot-Fol, 1957)	Capo Miseno, Bay of Naples, Italy	udoteal (**23**)	dietary from the green alga *Udotea petiolata*	[3,40]
		*Elysia halimedae* (Macnae 1954) (accepted as *Elysia pusilla* (Bergh, 1871))	Agat Bay, Guam	halimedatetracetate alcohol (**25**)	dietary (chemical modification of halimedatetraacetate (**24**) from *Halimeda mucroloba* Decaisne (Carlson and Hoff, 1978))	[52]
		*Elysia rufescens* (Pease, 1871)	Hawaii	kahalalide F (**26**) and isokahalalide F (**27**)	dietary (algae *Bryopsis pennata*; might be of bacterial origin, with *Mycoplasma* spp. and *Vibrio* spp. as possible producers)	[46,53,55,57]
		*Elysia ornata* (Swainson, 1840)	coasts of Okha (India)	kahalalide F (**26**) and other kalahalides	dietary (algae *Bryopsis pennata*; might be of bacterial origin, with *Mycoplasma* spp. and *Vibrio* spp. as possible producers)	[46,48]
		*Elysia grandifolia* (Kelaart, 1858)	Gulf of Mannar and Palk Bay, Rameswaram, India at 1 to 2 m depth.	kahalalide F (**26**) and other kalahalides	dietary (slugs were feeding on algae *Bryopsis plumosa (Hudson)*; might be of bacterial origin, with *Mycoplasma* spp. and *Vibrio* spp. as possible producers)	[46,54]
		*Thuridilla hopei* (Vérany, 1853)	Italy	thuridillins, e.g., thuridillin A (**28**)	de novo, with precursor derived from algae *Pseudochlorodesmis furcellata* (**30**)	[58,60]
		*Thuridilla splendens* (Baba, 1949)	Australia	thuridillins	de novo, with precursor derived from algae *P. furcellata* **30**)	[59,60]
		*Thuridilla hopei* (Vérany, 1853)	Italy	thuridillin-related aldehydes, e.g., nor-thuridillinal (**29**)	de novo, with precursor derived from algae *P. furcellata* (**30**)	[58,60]
Cephalaspidea	Aglajidae Pilsbry, 1895 (1847)	*Philinopsis speciosa* (Pease, 1860)	Hawaii	Kulolide-1 (**31**), kulolide-2 (**32**), kulolide-3 (**33**), kulokainalide-1 (**34**), kulokahilide-1 (**35**), kulokahilide-1 (**36**) as well as tolytoxin 23-acetate (**37**)	dietary (cyanobacteria; transfer most likely mediated via herbivorous molluscs like *Stylocheilus longicauda* and *Dolabella auricularia* which in turn were readily accepted by *P. speciosa* in feeding experiment)	[61,62,63,64]
		*Navanax inermis* (J. G. Cooper, 1862)	Pacific	navenones A-C (**39**-**41**), isopulo’upone (**42**), 5,6-dehydroaglajne-3 (**43**)	dietary (*Bulla* species, e.g., *Bulla gouldiana*)	[67,68,70]
	Gastropteridae Swainson, 1840	*Sagaminopteron psychedelicum* (Carlson and Hoff, 1974)	Guam	3,5 dibromo-2-(2′,4′-dibromo-phenoxy)phenol (**47**)	dietary (sponge *Dysidea granulosa*)	[71,72]
		*Sagaminopteron nigropunctatum* (Carlson and Hoff, 1973)	Guam	3,5 dibromo-2-(2′,4′-dibromo-phenoxy)phenol (**47**)	dietary (sponge *Dysidea granulosa*)	[71,73]
	Haminoeidae Pilsbry, 1895	*Haminoea cyanomarginata* (Heller and Thompson, 1983)	Gulf of Corinth (Greece)	brominated tetrahydropyran (**48**)	dietary (Western Australian sponge *Haliclona* sp. Grant, 1841)	[73]
		*Haminoea cymbalum* (Quoy and Gaimard, 1832)	Indian coasts	brominated tetrahydropyran (**48**)	dietary (sponge)	[73]
		*Haminoea cymbalum* (Quoy and Gaimard, 1832)	Guam	kumepaloxane (**49**)	dietary (sponge)	[74]
		*Haminoea* species	Naples (Italy)	haminol A–C (**50**–**52**), and haminol 1–6 (**53**–**58**	de novo	[75,76]
		*Haminoea fusari* (Alvarez, Garcia and Villani, 1993)	Naples (Italy)	polypropionates, haminol 1–6 (**53**–**58**)	de novo	[77]
		*Haminoea orbignyana* (Férussac, 1822)	Naples (Italy)	haminol 1 and 2 (**53**–**54**)	de novo synthesis, shown by feeding study	[4]
Pleurobranchomorpha	Pleurobranchidae Gray, 1827	*Pleurobranchus forskalii* (Rüppell and Leuckart, 1828)	Manado, Indonesia	keenamide A (**59**)	dietary (presumable cyanobacterial origin)	[78]
		*Pleurobranchus forskalii* (Rüppell and Leuckart, 1828)	Ishigaki Island, Japan	cycloforskamide (**60**)	dietary (sponge with associated cyanobacteria) or symbiotic cyanobacteria	[79]
		*Pleurobranchus forskalii* (Rüppell and Leuckart, 1828)	Ishigaki Island, Japan	ergosinine (**61**)	dietary (ascidian and/or endophytic fungi)	[81]
		*Pleurobranchus forskalii* (Rüppell and Leuckart, 1828)	Philippines	chlorolissoclimide (**62**) and dichlorolissoclimide **63**)	dietary (*Lissoclinum* species of ascidian)	[83,84]
		*Pleurobranchus. albiguttatus* (Bergh, 1905)	Philippines	chlorolissoclimide (**62**), dichlorolissoclimide (**63**) and 3β-hydroxychlorolissoclimide (**64**)	dietary (*Lissoclinum* species of ascidian)	[83,84]
Nudibranchia Anthobranchia	Aegiridae P. Fischer, 1883	*Notodoris citrina* (Bergh, 1875)	Gulf of Eilat, The Red Sea	naamidine A (**65**), isonaamidine-A (**66**)	dietary (sponge *Leucetta chagosensis* Dendy, 1913)	[85,86,87,88]
		*Notodoris gardineri* (Eliot, 1906)	Philippines	isonaamidine-A (**66**), dorimidazole-A (**67**)		[88,89]
		*Notodoris gardineri* (Eliot, 1906)	Great Barrier Reef	clathridine (**68**)	dietary (sponge)	[90,92,93]
		*Notodoris gardineri* (Eliot, 1906)	Papua New Guinea	clathridine (**68**)	dietary (sponge *Clathrina clathrus* Schmidt, 1864)	[91,92,93]
	Chromodorididae Bergh, 1891	*Ceratosoma amoenum* (Cheeseman, 1886)	Great Barrier Reef	allolaurinterol (**69**)	dietary (origin could be red algae, e.g., of the genus Laurencia; **69** also found in cyanobacteria, via herbivorous sea slugs)	[94,95,96]
		*Ceratosoma trilobatum* (J.E. Gray, 1827)	South China Sea Coast	(−)-furodysinin (**70**)	dietary (sponge)	[97]
		*Ceratosoma gracillimum* (Semper in Bergh, 1876)	South China Sea Coast	(−)-furodysinin (**70**)	dietary (sponge)	[97]
		*Ceratosoma gracillimum* (Semper in Bergh, 1876)	South Coast of Hainan Island	(−)-furodysinin (**70**), nakafuran-9 (**71**)	dietary (sponge)	[97]
		*Chromodoris lochi* (Rudman, 1982)	Fiji	latrunculin A (**72**)	dietary, **72** in *Spongia (=Cacospongia) mycofijiensis,* but could be produced by as yet uncultivated microorganism	[98,102]
		*Chromodoris lochi* (Rudman, 1982)	Vanuatu	mycothiazole (**74**)	dietary, sponges, but could be produced by as yet uncultivated microorganism	[103,104]
		*Chromodoris lochi* (Rudman, 1982)	Indonesia	laulimalide (syn fijianolide B) (**75**), isolaulimalide (syn fijianolide A) (**76**)	dietary, sponges, but could be produced by as yet uncultivated microorganism	[107,108,109]
		*Chromodoris hamiltoni* (Rudman, 1977)	South Africa	Lantrunculin A (**72**) and B (**73**)	dietary (sponge)	[100,102]
		*Chromodoris elisabethina* (Bergh, 1877)	Queensland, Australia	Lantrunculin A (**72**) and B (**73**)	dietary (sponge)	[101,102]
		*Chromodoris magnifica* (Quoy and Gaimard, 1832)	Queensland, Australia	Lantrunculin A (**72**) and B (**73**)	dietary (sponge)	[101,102]
		*Chromodoris kuiteri* (Rudman, 1982)	Queensland, Australia	Lantrunculin A (**72**) and B (**73**)	dietary (sponge)	[101,102]
		*Chromodoris annae* (Bergh, 1877)	Queensland, Australia	Lantrunculin A (**72**) and B (**73**)	dietary (sponge)	[101,102]
		*Chromodoris quadricolor* (Rüppell and Leuckart, 1830)	Red Sea	Lantrunculin A (**72**) and B (**73**)	dietary (sponge)	[101,102]
		*Chromodoris inorata* (Pease, 1871) (accepted as *Chromodoris aspersa* (Gould, 1852))	Japan	inorolide A (**77**), B (**78**), C (**79**) and various scalaranes		[110]
		*Chromodoris luteorosea* (Rapp, 1827) (accepted as *Felimida luteorosea* (Rapp, 1827))	Spain	norrisolide (**80**), polyrhaphin C (**81**), chelonaplysin C (**82**), luterosin (**83**), macfarlandin A (**84**),	dietary (sponge)	[111]
		*Chromodoris macfarlandi* (Cockerell, 1901) (accepted as *Felimida macfarlandi* (Cockerell, 1901))	California, USA	macfarlandines A–E (**84**–**88**)	dietary (sponge, structures related to compounds from *Aplysilla sulphurea*)	[111,112,113]
		*Chromodoris sinensis* (Rudman, 1985) (accepted as *Goniobranchus sinensis* (Rudman, 1985))	South China Sea	Aplyroseol-2 (**89**)	dietary (sponge, structures related to compounds from *Aplysilla* sp.)	[97]
		*Chromodoris reticulata* (Quoy and Gaimard, 1832) (accepted as *Goniobranchus reticulatus* (Quoy and Gaimard, 1832))	Australia	Aplyroseol-2 (**89**) and other diterpenes	dietary (sponge, structures related to compounds from *Aplysilla* sp.)	[115]
		*Chromodoris maridadilus* (Rudman, 1977) (accepted as *Hypselodoris maridadilus* (Rudman, 1977))	Hawaii	nakafuran-9 (**71**), nakafuran-8 (**90**)	dietary (sponge *Dysidea fragilis*)	[116]
		*Glossodoris atromarginata* (Cuvier, 1804) (accepted as *Doriprismatica stellata* (Cuvier, 1804))	Sri Lanka, Australia, India	furanoditerpenoid and scalarane type, structural variants of these metabolites (differences due to diff. sponge prey); spongiadiol (**91**), spongiadiol diacetate (**92**), epispongiadiol (**93**), 12-deacetoxy-12-oxodeoxoscalarin (**94**), heteronemin (**95**), mooloolabene D (**96**)	dietary (sponge, e.g., *Spongia* sp. (former *Hyatella intestinales* (Lamarck, 1814)), *Hyrtios erectus* (Keller, 1889) and *Hyrtios* sp.)	[117,118,119,120,121,122,123,124,125,126,127,128,129,130,131]
		*Glossodoris dalli* (Bergh, 1879) (accepted as *Felimida dalli* (Bergh, 1879))	Natural Park of Osa Ballena (Costa Rica)	homoscalarane and scalarane compounds	probably dietary from sponges	[132]
		*Glossodoris sedna* (Ev. Marcus and Er. Marcus, 1967) (accepted as *Doriprismatica sedna* (Ev. Marcus and Er. Marcus, 1967))	Natural Park of Osa Ballena (Costa Rica)	12-deacetyl-23-acetoxy-20-methyl-12-*epi*scalaradial (**97**)	probably dietary from sponges	[132]
		*Glossodoris rufomarginata* (Bergh, 1890)	Hainan Island in the South China Sea	homoscalarane and scalarane compounds	probably dietary from sponges	[124]
		*Glossodoris pallida* (Rüppell and Leuckart, 1830)	China and Guam	homoscalarane and scalarane compounds, different pattern at different location	probably dietary from sponges	[119]
		*Glossodoris vespa* (Rudman, 1990)	Eastern Australia	homoscalarane and scalarane compounds	probably dietary from sponges	[119]
		*Glossodoris averni* (Rudman, 1985) (accepted as *Ardeadoris averni* (Rudman, 1985))	Eastern Australia	homoscalarane and scalarane compounds	probably dietary from sponges	[119]
		*Goniobranchus obsoletus* (Rüppell and Leuckart, 1830)	Japan	most bioactive: dorisenones A (**98**), B (**99**), D (**100**), 11β-hydroxyspongi-12-en-16-one (**101**), spongian-16-one (**102**)	dietary (sponge *Spongionella* sp.)	[133]
		*Goniobranchus splendidus* (Angas, 1864)	Australia	spongian-16-one (**102**), aplytandiene-3 (**103**), aplysulfurin (**104**) and aplyroseol-2 (**89**), the gracilins A (**105**), B (**106**), C (**107**), G (**108**), M (**109**))	dietary (sponge *Spongionella* sp.)	[134,135,203]
		*Hypselodoris infucata* (Rüppell and Leuckart, 1830)	Hawaii	nakafuran-8 (**90**) and nakafuran-9 (**71**)	dietary (sponge *Dysidea fragilis* (Montagu, 1814))	[116,138]
	Dendrodorididae O’Donoghue, 1924 (1864)	*Dendrodoris limbata* (Cuvier, 1804)		olepupuane (**116**) and polygodial (**117**)	de novo	[147,150,151,152]
	Discodorididae Bergh, 1891	*Halgerda aurantiomaculata* (Allan, 1932)	Japan, Australia	zooanemonin (**118**) and esmodil (**119**)	dietary (sponge, anemone *Anemonia sulcate*)	[153]
		*Halgerda gunnessi* Fahey and Gosliner, 2001	Japan, Australia	investigated, but no compounds found		[153]
		*Halgerda rubicunda* (Baba, 1949) (accepted as *Sclerodoris rubicunda* (Baba, 1949))	Japan, Australia	investigated, but no compounds found		[153]
		*Halgerda theobroma* (Fahey and Gosliner, 2001)	Japan, Australia	investigated, but no compounds found		[153]
		*Halgerda willeyi* (Eliot, 1904)	Japan, Australia	investigated, but no compounds found		[153]
		*Paradoris indecora* (Bergh, 1881)	Spain, Italy	variabilin (**120**)	dietary (sponge, e.g., *Ircinia* sp.)	[154]
		*Jorunna funebris* (Kelaart, 1859)	India	jorumycin (**121**)	from structural similarity to ecteinascidin 743 (**122**) bacterial origin	[155,156,157]
	Hexabranchidae Bergh, 1891	*Hexabranchus sanguineus* (Rüppell and Leuckart, 1830)	Hawaii, Japan	Trisoxazole macrolides, i.e., ulapualide A (**123**) and B (**124**), halichondramides (**126**–**129**), kabiramide A–E (**125**, **130**–**133**),	dietary, trisoxazole macrolides isolated from different sponges from the genera *Halichondria*, *Mycale*, *Jaspis* and *Pachastrissa*. halichondramides (**126**–**129**) isolated from sponge *Halichondria* sp.	[156,157,158,159,160,161,162,163,164,165,166,167,168]
	Phyllidiidae Rafinesque, 1814	*Phyllidia coelestis* (Bergh, 1905)	Thailand	1-formamido-10(1,2)-abeopupukeanane (**134**), 2-formamidopupukeanane (**135**)	dietary (sponge)	[176,177]
		*Phyllidia ocellata* (Cuvier, 1804)	Japan	cavernothiocyanate (**136**), 10α-isocyano-4-amorphene (**137**), axisonitrile-3 (**138**), and 7-isocyano-7,8-dihydro-α-bisabolene (**139**)	dietary ((**136**–**139**) from sponge *Acanthella* cf. *cavernosa*)	[179]
		*Phyllidia ocellata* (Cuvier, 1804)	Australia	2-isocyanoclovene (**140**), 2-isocyanoclovane (**141**), 1-isothiocyanatoepicaryolane (**142**), 4,5-epi-10-isocyanoisodauc-6-ene (**143**)	dietary (sponges)	[180]
		*Phyllidia varicosa* (Lamarck, 1801)	Hawaii	9-isocyanopupukeanane (**144**), 2-isocyanopupukeanane (**145**)	dietary, sponge *Ciocalypta* sp. (ex. *Hymeniacidon* sp.)	[182,183]
		*Phyllidia varicosa* (Lamarck, 1801)	Indonesia	9-isocyanopupukeanane (**144**), epimeric 9-thiocyanato-pupekeanane (**147**, **148**)	dietary, sponge *Ciocalypta* sp. (ex. *Hymeniacidon* sp.)	[186]
		*Phyllidia varicosa* (Lamarck, 1801)	Sri Lanka	3-isocyanotheonellin (**146**)	dietary (sponge)	[185]
		*Phyllidiella rosans* (Bergh, 1873)	Japan	9-isocyanopupukeanane (**144**)	dietary (sponge)	[184]
		*Phyllidiella pustulosa* (Cuvier, 1804)	Japan	axisonitrile-3 (**138**), unnamed molecule with isocyano group (**155**) and substituted axinisothiocyanate K derivative	dietary (sponge)	[190]
		*Phyllidiella pustulosa* (Cuvier, 1804)	China	3-isocyanotheonellin (**146**), amphilectene (**152**), kalihinol-A (**153**), kalihinol-E (**154**)	dietary (sponges, due to very similar compounds present in both samples)	[175]
		*Phyllidiella pustulosa* (Cuvier, 1804)	Vietnam	9-isocyanopupukeanane (**144**) and its C-9 epimer	dietary, based on the terpenes obtained the authors concluded that the mollusk feeds on sponges of the genera Acanthella, Halichondria, Axinella and Axinyssa	[189]
		*Phyllidiella pustulosa* (Cuvier, 1804)	Fiji	axisonitrile-3 (**138**), 10-isothiocyano-4-cadinene (**150**)/10-thiocyanato-4-cadinene (**151**)	sponge *Phakellia carduus,* due to very similar secondary metabolites present in both samples	[187,188]
	Polyceridae Alder and Hancock, 1845	*Tambja abdere* (Farmer, 1978)	West coast of America	tambjamines A–D, tambjamines A (**156**), C (**157**)	dietary (bryozoan *Sessibugula translucens*)	[191]
		*Tambja eliora* (Er. Marcus and Ev. Marcus, 1967)	West coast of America	tambjamines A-D, tambjamines A (**156**), C (**157**)	dietary (bryozoan *Sessibugula translucens*)	[191]
		*Roboastra tigris* (Farmer, 1978)	Gulf of California	tambjamines A-D, tambjamines A (**156**), C (**157**)	dietary (bryozoan *Bugula neritida*)	[191]
		*Nembrotha* species	Micronesia	tambjamines A (**156**), C (**157**), E (**158**), F (**159**), the tambjamine aldehyde (**160**) and the blue tetrapyrrol (**161**)	dietary (ascidian *Atapozoa* sp.)	[138,192]
		*Nembrotha cristata* (Bergh, 1877)	Ant Atoll	tambjamines A (**156**), C (**157**), E (**158**), F (**159**), the tambjamine aldehyde (**160**) and the blue tetrapyrrol (**161**)	dietary (ascidian *Atapozoa* sp.)	[138,192]
		*Nembrotha kubaryana* (Bergh, 1877)	Sumilon Island, the Philippines	tambjamines C (**157**), E (**158**) and the blue tetrapyrrol (**161**)	dietary (ascidian *Atapozoa* sp.)	[138,192]
		*Nembrotha* sp.	Apo Islands, the Philippines	tambjamines C (**157**), E (**158**), F (**159**), and the tambjamine aldehyde (**160**)	dietary (ascidian *Atapozoa* sp.)	[192]
		*Polycera atra* (MacFarland, 1905)	Torrey Pines artificial reef	bryostatins, e.g., bryostatin 1 (**162**)	dietary (bryozoan *Bugula neritina*)	[193,194,195]
Subclade Cladobranchia	Facelinidae Bergh, 1889	*Phyllodesmium briareum* (Bergh, 1896)	not known	brianthein W (**110**) and excavatolide C (**111**)	dietary (coral *Briareum* sp.)	[1]
		*Phyllodesmium magnum* (Rudman, 1991)	not known	cembrane diterpenes, e.g., 11-episinulariolide acetate (**112**)	dietary, e.g., *Sinularia* spp., *Capnella* sp.	[1]
	Flabellinidae Bergh, 1889	*Flabellina exoptata* (Gosliner and Willan, 1991)	not known	hormarin (**113**)	dietary	[139,141]
		*Flabellina ischitana* (Hirano and Thompson, 1990)	not known	hormarin (**113**)	dietary	[139,141]
		*Flabellina pedata* (Montagu, 1816)	not known	hormarin (**113**)	dietary	[139,141]
		*Flabellina affinis* (Gmelin, 1791)	not known	hormarin (**113**)	dietary	[139,141]
	Proctonotidae Gray, 1853	*Janolus cristatus* (Delle Chiaje, 1841)	Mediterranean	janolusimide (**114**)	dietary (bryozoa)	[139,141]
